# Effects of Deficit Irrigation and Huanglongbing on Sweet Orange Trees

**DOI:** 10.3389/fpls.2021.731314

**Published:** 2021-10-15

**Authors:** Jefferson Rangel da Silva, Rodrigo Marcelli Boaretto, Jéssica Aparecida Lara Lavorenti, Bruna Castriani Ferreira dos Santos, Helvecio Della Coletta-Filho, Dirceu Mattos

**Affiliations:** Centro de Citricultura Sylvio Moreira, Instituto Agronômico (IAC), Cordeirópolis, Brazil

**Keywords:** starch metabolism, photosynthesis, leaf respiration, photorespiration, leaf water potential, greening, oxidative stress

## Abstract

This study addresses the interactive effects of deficit irrigation and huanglongbing (HLB) infection on the physiological, biochemical, and oxidative stress responses of sweet orange trees. We sought to answer: (i) What are the causes for the reduction in water uptake in HLB infected plants? (ii) Is the water status of plants negatively affected by HLB infection? (iii) What are the key physiological traits impaired in HLB-infected plants? and (iv) What conditions can mitigate both disease severity and physiological/biochemical impairments in HLB-infected plants? Two water management treatments were applied for 11 weeks to 1-year-old-trees that were either healthy (HLB–) or infected with HLB (+) and grown in 12-L pots. Half of the trees were fully irrigated (FI) to saturation, whereas half were deficit-irrigated (DI) using 40% of the water required to saturate the substrate. Our results demonstrated that: reduced water uptake capacity in HLB+ plants was associated with reduced root growth, leaf area, stomatal conductance, and transpiration. Leaf water potential was not negatively affected by HLB infection. HLB increased leaf respiration rates (*ca*. 41%) and starch synthesis, downregulated starch breakdown, blocked electron transport, improved oxidative stress, and reduced leaf photosynthesis (*ca*. 57%) and photorespiration (*ca*.57%). Deficit irrigation reduced both leaf respiration (*ca*. 45%) and accumulation of starch (*ca*.53%) by increasing maltose (*ca*. 20%), sucrose, glucose, and fructose contents in the leaves, decreasing bacterial population (*ca*. 9%) and triggering a series of protective measures against further impairments in the physiology and biochemistry of HLB-infected plants. Such results provide a more complete physiological and biochemical overview of HLB-infected plants and can guide future studies to screen genetic tolerance to HLB and improve management strategies under field orchard conditions.

## Introduction

The most important problem in citrus production worldwide is the bacterial disease huanglongbing (HLB; *syn*. citrus greening), presumably caused by the bacterium *Candidatus* Liberibacter asiaticus (*C*las; Wang and Trivedi, 2013) that triggers a cascade of events, causing phloem dysfunction, cellular collapse, and accumulation of carbohydrates in leaves (Cimò et al., 2013). Although the dysfunction caused by HLB in citrus has been associated with the accumulation of starch in both chloroplasts and sieve elements of infected trees (Etxeberria et al., 2009; Fan et al., 2010; Folimonova and Achor, 2010; Koh et al., 2012; Aritua et al., 2013; Johnson et al., 2014; Mattos-Jr et al., 2020), processes associated with starch synthesis/breakdown have not been fully elucidated in HLB plants (Etxeberria et al., 2009; Gibon et al., 2009; Fan et al., 2010). Understanding the changes in carbohydrate metabolism may enable improvements in management strategies that could mitigate the deleterious effects of the disease on citrus orchards (Mattos-Jr et al., 2020).

Citrate has been reported as the likely main source of energy for the phloem-inhabiting pathogen/bacteria *Clas* (Cruz-Munoz et al., 2018). In this case, leaf respiration (*R*) as a key component of plant growth (Ayub et al., 2014), if leading to a negative carbon balance, could have a central role in plant susceptibility to HLB. HLB also damages photosynthetic capacity, which can be associated with the production of reactive oxygen species (ROS), oxidative stress, and components of H_2_O_2_ detoxification (Martinelli and Dandekar, 2017).

The plugging of sieve pores by starch granules was suggested to inhibit the transport of photoassimilates to sink tissues (Etxeberria and Narciso, 2012), restricting root growth and, hence, the uptake of water and nutrients (Johnson et al., 2014; Kadyampakeni et al., 2014; Hamido et al., 2016; Mattos-Jr et al., 2020; Silva et al., 2020). Any degree of water stress in trees is considered to exacerbate the deleterious effects of HLB, since maintaining daily irrigation is taken as an important management practice for HLB-affected trees, and reduces tree water stress and improve tree water use (Kadyampakeni et al., 2014; Kadyampakeni and Morgan, 2017). Nonetheless, only a few studies have addressed the water status of plants infected with *C*Las (Kumar et al., 2018), and there have been no studies focused on the effects of HLB under reduced water availability conditions. It is important to note that the water economy usually drives the main focus of deficit irrigation in agricultural production (Fereres and Soriano, 2007), whereas we verify a lack of understanding of mechanisms with which plants respond to such management and increase crop production (Chai et al., 2016).

We considered the interactive effects of deficit irrigation and HLB infection on a variety of important physiological and growth traits in citrus trees. Elucidating the responses of HLB-infected plants under deficit irrigation conditions can provide insights into key physiological processes modified by the disease and guide further studies in finding some degree of tree tolerance to the disease. Furthermore, the knowledge of the water status of HLB-infected plants can lead toward optimum irrigation management to conserve water and reduce nutrient losses (Kadyampakeni et al., 2014; Hamido et al., 2017; Kadyampakeni and Morgan, 2017). Thus, we sought to answer (i) what are the causes of the reduction in water uptake in HLB-infected plants? (ii) Is the water status of plants negatively affected by HLB infection? (iii) What are the key physiological traits impaired in HLB-infected plants? and (iv) what conditions can mitigate both disease severity and physiological/biochemical impairments in HLB-infected plants?

## Materials and Methods

### Plant Material, Growth Conditions, Bacterial Infection, and Water Management Treatments

This study was conducted in a greenhouse at the Centro de Citricultura Sylvio Moreira, in Cordeirópolis, São Paulo, Brazil. The north-south-oriented greenhouse was covered with 150-μm thick transparent plastic and closed on all sides with an anti-aphid screen, with natural fluctuations of climatic conditions. Climate variables were monitored throughout the experiment using a weather station (Vantage Pro2; Davis Instruments, Hayward, CA, United States). Average, maximum, and minimum solar radiation in μmol m^−2^ s^−1^ were 914.9 ± 154.7, 1,664.5 ± 372.3, and 155.7 ± 76.7, respectively. Average, maximum, and minimum air temperature in °C were 22.6 ± 3.8, 31.8 ± 4.9, and 15.4 ± 3.2, respectively. Average, maximum, and minimum relative humidity (%) were 67.6 ± 9.5, 89.1 ± 6.5, and 40.6 ± 11.4, respectively.

Twelve-month-old sweet orange trees cv. Valência [*Citrus sinensis* (L.) Osbeck] grafted on citrumelo “Swingle” rootstock [*Citrus paradisi* Macfad. x *Poncirus trifoliata* (L.) Raf.] were obtained from a commercial nursery and transplanted into 12-L pots filled with a well-drained commercial pine-bark-based substrate (Terra do Paraíso®). The pots were wrapped in a reflective aluminized blanket to avoid substrate over-heating. At transplant, each tree had a single stem and nine leaves.

Fifteen days later, the trees were separated into two groups and either (i) bud grafted onto opposite sides of the main stem with two buds obtained from micrografted plants (healthy, –) or (ii) bud grafted onto opposite sides of the main stem with two budwoods obtained from *C*Las source plants maintained in our laboratory and previously tested for the presence of bacteria by real-time quantitative PCR (qPCR) (infected plants, +). In both groups of the plants, buds with lignified tissue presenting a length of *ca*. 4 cm were used.

The plants were pruned to allow for uniform growth of two branches per plant (first vegetative flush) 37 days after transferring them to the 12-L pots. Leaves (one per branch) closest to the main stem were tagged to measure central vein length. Three Soil Plant Analysis Development (SPAD) values were averaged in each leaf used for central vein measurements, using the SPAD-502 Chlorophyll Meter (Minolta Co. Ltd, Osaka, Japan). The number of leaves on each branch was also evaluated. Such morphological measurements were performed once a week for 13 weeks until the first vegetative flush of growth was considered completely expanded (127 days after transferring plants to the 12-L pots). At this point, six leaves of the first vegetative flush per individual plant (of a total of 16 plants) were sampled and used for DNA extraction and *C*Las quantification by qPCR as described below. One hundred twenty-seven days after transferring the plants to the 12 L pots, cycle threshold values (C_T_) for HLB+ shoots were determined as indirect measures of *C*Las titer and were *ca*. 22.51 ± 0.55, showing that *C*Las inoculation was efficient (Ammar et al., 2011; Coletta-Filho et al., 2014; Canale et al., 2017).

**Figure 1:**
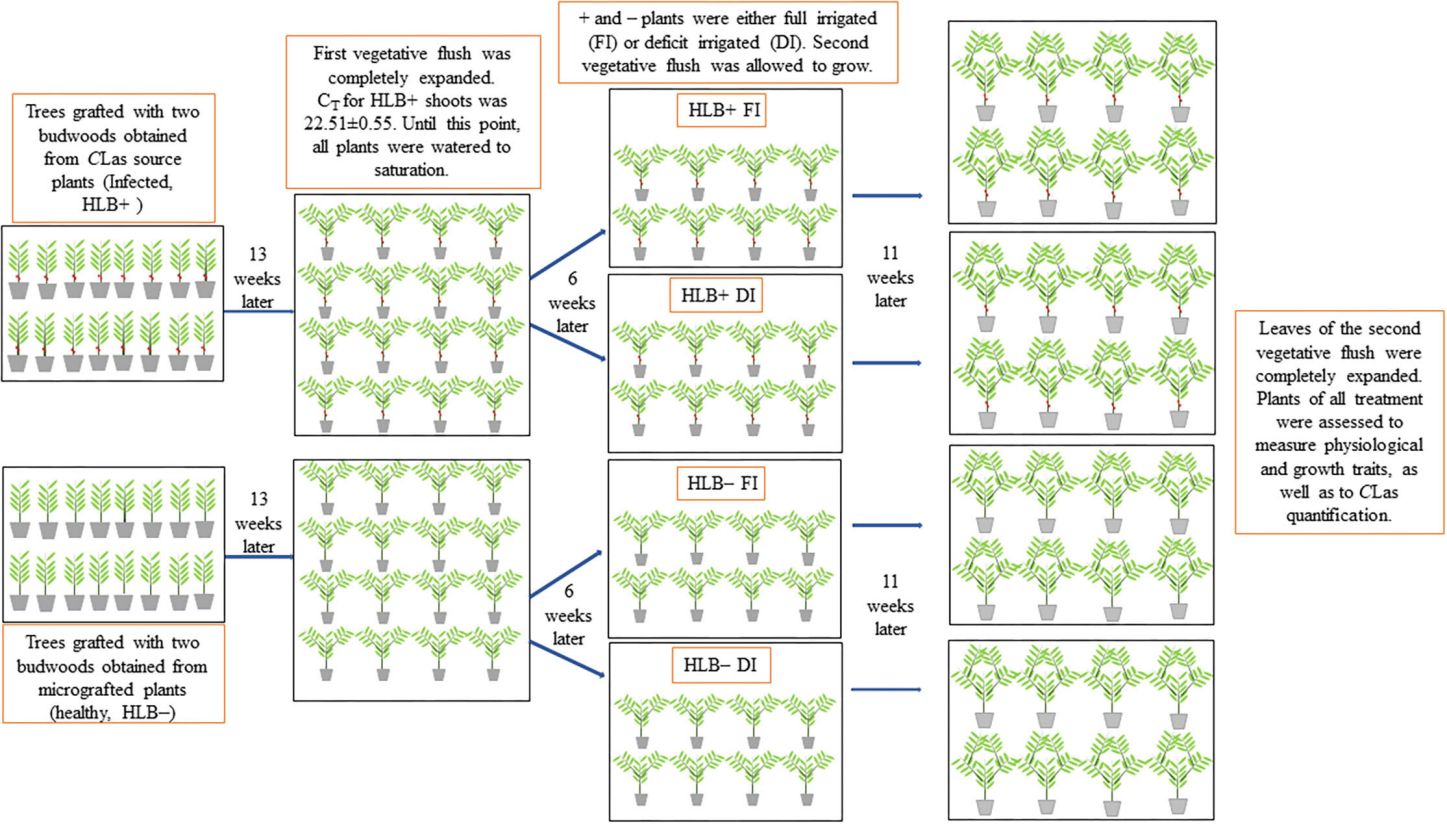
Schematic representation of the experiment. Young citrus trees were separated into two groups and either (i) bud grafted with two buds obtained from micrografted plants (healthy, –) or (ii) bud grafted with two budwoods obtained from *C*Las source plants (infected plants, +). Two branches were allowed to grow per plant (first vegetative flush). Thirteen weeks later, the first vegetative flush was completely expanded, and HLB+ shoots presented a cycle threshold value (C_T_) of *ca*. 22.5. Six-weeks later, half of the trees were fully irrigated (FI) to saturation, and the other half were deficit-irrigated (DI) using 40% of the water required to saturate the substrate. Thereafter, the second vegetative flush was allowed to grow for 11 weeks. At this point, plants of all the treatments were assessed to measure physiological and growth traits and quantify *C*Las.

All the pots were watered daily until saturation for 171 days. Thereafter (6 weeks after *C*Las quantification), both + and – plants were exposed to one of two water management treatments: (1) fully irrigated (FI): plants were irrigated 3 days a week to saturation; (2) deficit-irrigated (DI): plants were irrigated 3 days a week using 40% of the water applied to the respective FI treatment. The 40% deficit irrigation level was selected given the fact that we expected to induce mild physiological responses in the plants, as demonstrated by Pedroso et al. (2014). There were eight replicate trees in each of the four treatments (Figure 1). To determine the amount of water required to saturate the substrate, each FI pot was irrigated with a known volume of water to saturation. Outflow from the pots was collected in a graduated cylinder, and watering was stopped when water was observed to be leaking from the bottom of the pots. The difference between the volume of the drained water and the amount of water applied was the volume of water needed to saturate the substrate. The amount of water required to irrigate the DI plants was calculated as:


(1) $$\begin{array}{l} V_{DI}\;=\;\frac{V_{FI}\;.\;40\;}{100} \end{array}$$

where *V*_*DI*_ is the volume of water required to irrigate the DI(+)- and DI(–)-treated plants, and *V*_*FI*_ is the average volume of water applied to either FI+ or FI–, respectively. The total water amount applied to each treatment was recorded. Volumetric substrate moisture was monitored every week at a depth of 0.15 m using a soil moisture probe (model number MO750; Extech Instruments, Nashua, NH, United States). Three values of volumetric soil moisture were averaged in each pot.

All the plants were fertilized monthly with a complete nutrient solution containing in g per pot: 3.4 Ca(NO_3_)_2_.4H_2_O; 0.85 MgSO_4_.7H_2_O; 1.95 NH_4_NO_3_; 1.1 KH_2_PO_4_; 0.5 of NH_4_SO_4_; 0.5 of CaCl_2_; 0.01 of NaMoO_4_; 0.01 g of ZnSO_4_; 0.01 of MnSO_4_; 0.01 of CuSO_4_; 0.02 of H_3_BO_3_; and 0.1 Fe-EDDHA containing 6% Fe. The volume and concentration of the nutrient solution were continuously adjusted according to each water management treatment for all the plants to always receive the same amount of nutrients, regardless of the volume of nutrient solution applied to each treatment.

A day after initiating water management treatments (172 days after transferring the plants to the 12-L pots), another two branches (second vegetative flush) were allowed to grow, one from the tip of each branch of the first vegetative flush (Figure 1). The same morphological measurements to characterize the first vegetative flush were performed in the second vegetative flush of growth. Therefore, one leaf per branch of the second vegetative flush that was most proximate to the first vegetative flush was tagged, so central vein length and SPAD measurements could be taken, as described above. Morphological measurements of the second vegetative flush were performed once a week for 11 weeks (225 days after transferring the plants to the 12-L pots) when all the leaves had completely expanded. At this point, the plants of all the treatments were assessed to measure a variety of physiological and growth traits, as well as to diagnose and quantify *C*Las in both fine roots and petiole of the leaves from the first and second vegetative flushes.

### Leaf Gas Exchange

Leaf gas exchange measurements were taken on leaves of the second vegetative flush that were most proximate to the first vegetative flush. Light response curves of net photosynthetic rate (*A*_*net*_) using a Li-Cor 6,800 (Li-Cor Inc., Lincoln, NE, United States) portable photosynthesis system were obtained at 08:00 and 11:00 a.m. and as described in Shapiro et al. (2004) taking into consideration the precautions of Pons and Welschen (2002). The system incorporated a CO_2_ controller that was used to set the CO_2_ concentration inside the leaf cuvette to 400 μl L^−1^. A 6 cm^2^-cuvette was fitted with a red-blue light source. The *A*_*net*_, stomatal conductance (*g*_*s*_) and transpiration rate (*E*) at 800 μmolm^−2^ s^−1^ were determined from the light response curves with 24 levels of photosynthetic photon flux density (PPFD): 1,500, 1,200, 800, 500, 200, 100, 90, 80, 70 μmol m^−2^ s^−1^ and every five units between 70 and 0 μmol m^−2^ s^−1^. The 800-μmol m^−2^ s^−1^ light level was previously determined to be the light saturation point for citrus trees. The rate of respiration in the dark (*R*_*dark*_) was taken as the value of *A*_*net*_ at 0 μmol m^−2^ s^−1^ PPFD.

The rate of respiration in the light (*R*_*light*_) was estimated using the method originally described in Kok (1948), as the y-axis intercept of a first-order linear regression fitted to *A*_*net*_–irradiance plots to measurements made over the 25–65 μmol m^−2^ s^−1^ irradiance range. All gas exchange data were corrected for an increase in intercellular CO_2_ concentrations (*C*_*i*_) with a decrease in irradiance, which can result in reduced rates of photorespiration and increased rates of carboxylation (Villar et al., 1994). The correction was applied by adjusting the *R*_*light*_ through iteration to minimize the intercept of photosynthetic electron transport (*J*) as a function of irradiance (Kirschbaum and Farquhar, 1987). *J* was calculated according to Farquhar and von Caemmerer (1982):


(2) $$\begin{array}{l} J=\;\frac{\left[\left(4\left(A_{net}+\;R_{light}\right)\right)\left(C_{i}+2\Gamma^{*}\right)\right]}{\left(C_{i}-\;\Gamma *\right)}, \end{array}$$

where Γ^*^ is the CO_2_ compensation point in the absence of *R*_*light*_ (von Caemmerer and Farquhar, 1981; 38.6 at 25°C). The rates of oxygenation and carboxylation by ribulose-1,5-bisfosfato carboxilase oxygenase (Rubisco, EC 4.1.1.39 – *V*_*o*_ and *V*_*c*_, respectively) were calculated at 800 μmol m^−2^ s^−1^ (light saturation point), according to Farquhar and von Caemmerer (1982):


(3) $$\begin{array}{l} V_{c}=\;\frac{1}{3}\left[\left(\frac{J}{4}\right)+2\left(A_{net}+R_{light}\right)\right], \end{array}$$

(4) $$\begin{array}{l} V_{o}=\;\frac{2}{3}\left[\left(\frac{J}{4}\right)-\left(A_{net}+R_{light}\right)\right]. \end{array}$$

The effects of varying atmospheric [O_2_] or [CO_2_] on oxygenation (*V*_*o*_) and carboxylation (*V*_*c*_) at each intensity of light used were also calculated according to Farquhar and von Caemmerer (1982):


(5) $$\begin{array}{l} V_{c}=\;\frac{\left[CO_{2}\right]\;V_{cmax}}{\left[CO_{2}\right]+K_{c}\left(1+\frac{\left[O_{2}\right]}{K_{o}}\right)} \end{array}$$

and


(6) $$\begin{array}{l} V_{o}=\;\frac{\left[O_{2}\right]\;V_{omax}}{\left[O_{2}\right]+K_{o\;}\left(1+\frac{\left[CO_{2}\right]}{K_{c}}\right)}. \end{array}$$

Equations (5, 6) used the Rubisco kinetic constants (*K*_*c*_ = 404.9 μmol mol^−11^; *K*_*o*_ = 278.4 mmol mol^−1^) previously determined by Bernacchi et al. (2001) at 25°C, and calculated *V*_*cmax*_ as:


(7) $$\begin{array}{l} V_{cmax}=\;\frac{A_{net}-R_{light}}{\frac{\left[{CO}_{2}\right]-\Gamma^{*}}{\left[CO_{2}\right]+K_{c}\left(1+\frac{\left[O_{2}\right]}{K_{c}}\right)}}. \end{array}$$

Γ ^*^ (the CO_2_ compensation point in the absence of R_light_) depends on the Rubisco specificity factor, O_2_ partial pressure, and is calculated according to von Caemmerer and Farquhar (1981) for real leaf temperature:


(8) $$\begin{array}{l} \Gamma^{*}=\;\frac{0.5V_{omax}K_{c}\left[O_{2}\right]}{V_{cmax}K_{o}}. \end{array}$$

In this study, we used the specificity presented in Cousins et al. (2010), ambient O_2_ concentration, and temperature response function of Brooks and Farquhar (1985) to determine Γ^*^. The values of *V*_*o*_, *V*_*c*_, and *V*_*c*_*:V*_*o*_ ratio reported here were calculated at an ambient CO_2_ concentration.

All the gas exchange measurements were taken at a relative humidity of approximately 45% by manipulating the amount of air passing through a drying column prior to entering the leaf cuvette. Air temperature in the Li-Cor cuvette was set to 30°C for all the measurements to avoid any influence of leaf temperature on gas exchange variables. In addition, the flow rate was kept at 500 μmol m^−2^ s^−1^.

### Chlorophyll a Fluorescence and JIP Test Measurements

Fast chlorophyll induction kinetics (measured for 1 s), typically known as OJIP, was measured with the Li-Cor 6800 (Li-Cor Inc., Lincoln, NE, United States) portable photosynthesis system. The measurements were taken between 06:00 and 7:00 a.m. after dark-adapting the same leaves used for gas exchange measurements overnight. Reflective aluminized foil was used to wrap the leaves to avoid any light exposure before the measurements. An induction flash intensity of 16,000 μmol m^−2^ s^−1^ was used to yield minimal (F_0_) and maximal fluorescence (F_m_) from which the maximum quantum yield of primary photochemistry was derived (Genty et al., 1989) as *F*_*v*_*/F*_*m*_.

The JIP test equations (Strasser and Strasser, 1995; Strasser et al., 1995, 2004) were applied to calculate: the effective antenna size of an active reaction center (*RC*) (*ABS/RC*); the maximal trapping rate of PS II (*TR*_0_*/RC*); the electron transport in an active RC (*ET*_0_*/RC*); the effective dissipation of an active RC (*DI*_0_*/RC*); the electron transport probability (*ET*_0_*/TR*_0_); the quantum yield of electron transport (*ET*_0_*/ABS*); the number of photons absorbed by an excited photosystem II (*PSII*) cross-section (*ABS/CS*_0_); the maximal trapping rate in a *PSII* cross-section (*TR*_0_*/CS*_0_); the electron transport in a *PSII* cross-section (*ET*_0_*/CS*_0_); the fraction of active reaction centers per excited cross-section of the leaf (*RC/CS*_0_); and the performance index (*PI*, energy cascade processes from the first light absorption event until plastoquinone reduction). To present the data in spider plots with the same scale for all parameters, the values obtained for the HLB– FI plants were considered as the standard (=1), whereas the values of the other treatments were plotted as the relative change in relation to this standard, based on real values.

### Leaf Water Potential

Leaf water potential (Ψ) was measured immediately after leaf excision at pre-dawn (Ψ_*pd*_) and midday (Ψ_*md*_) according to Scholander et al., 1964, using a pressure chamber (model 1000; PMS Instrument Co., Albany, OR, United States). The drop (%) of Ψ_*md*_ in relation to Ψ_*pd*_ was also calculated (Ψ_*drop*_). The measurements were performed on leaves adjacent to those used for gas exchange analysis (leaves of the second vegetative flush of growth).

### Quantification of Starch and Soluble Sugars in Leaves

Three leaves from the second vegetative flush from each tree (*ca*. 100 mg) were harvested at midday and added into liquid N_2_ following the extraction of starch in 0.7 mol.L^−1^ perchloric acid as described by Delatte et al., 2006. The insoluble material was washed once with water and three times with 80% (v/v) ethanol to remove residual soluble glucans and pigments. Starch in the insoluble fraction was digested with α-amylase (EC 3.2.1.1; Megazyme, Bray, Ireland) and amyloglucosidase (EC 3.2.1.3; Megazyme, Bray, Ireland). Glucose equivalents were determined by an enzymatic reaction using hexokinase (EC 2.7.1.1; Roche, Basel, Switzerland) and glucose-6-phosphate dehydrogenase (EC 1.1.1.49; Roche, Basel, Switzerland), which converts NAD to NADH in an equimolar ratio as described by Smith and Zeeman (2006). The increase in the absorption spectrum at 340 nm (OD340) was assayed in a spectrophotometer (Multiskan Go; Thermo Fisher Scientific, Waltham, MA, United States).

The supernatant was used to determine the concentration of soluble sugars. Glucose, fructose, and sucrose were quantified enzymatically according to Viola and Davies (1992) with modifications described by Thalmann et al. (2016). Briefly, 10 μl of the samples were added to 183 ml of 50 mmol L^−1^ HEPES buffer (pH 7.5) containing 1 mmol L^−1^ATP, 1 mmol L^−1^ NAD, and 1 mmol L^−1^ MgCl_2_. To measure glucose, hexokinase (EC 2.7.1.1; Roche, Basel, Switzerland) and glucose 6-phosphate dehydrogenase (1.1.1.49; Roche, Basel, Switzerland) were used to convert glucose to 6-phosphogluconate with concomitant reduction of NAD to NADH, which was monitored spectrophotometrically (Multiskan Go; Thermo Fisher Scientific, Waltham, MA, United States) at 340 nm. Subsequently, phosphoglucoisomerase (EC 5.3.1.9; Roche, Basel, Switzerland) was added to determine the amount of fructose. Finally, invertase (EC 3.2.1.26; Sigma-Aldrich, St. Louis, MO, United States) was added to cleave sucrose into fructose and glucose. The further increase in OD340 represented sucrose.

Maltose was determined according to Revanna et al. (2013), with modifications described by Smirnova et al. (2017). A two-step assay was performed using a 96-well microplate. First, in a volume of 100 μl, the sample containing maltose, 100 mmol L^−1^ citrate-NaOH (pH 6.5), and 2 U maltase (a-glucosidase, EC 3.2.1.20; Megazyme, Bray, Ireland) was incubated for 30 min. The absorbance at 340 nm was measured at the end of the first step with a spectrophotometer (Multiskan Go; Thermo Fisher Scientific, Waltham, MA, United States). Subsequently, a total of 100 μl of a solution containing 100 mM Tricine-NaOH (pH 7.8), 5 mmol L^−1^ MgCl_2_, 1 mmol L^−1^ ATP, 1 mmol L^−1^ NADP^+^, 0.5 U hexokinase, and 0.25 U glucose 6-phosphate dehydrogenase was added. The increase in the absorption spectrum at 340 nm specific for NADH was measured again.

### Hydrogen Peroxide, Lipid Peroxidation, and Superoxide Dismutase (SOD, EC 1.15.1.1) Activity

For protein quantification and SOD activity, 0.4 g of fine leaf powder (from the second vegetative flush) was homogenized in 2 ml of 100 mmol L^−1^ potassium phosphate buffer (pH 7.5), with 3 mmol L^−1^ dithiothreitol, 1 mmol L^−1^ EDTA, and 4% (w/v) PVPP (Capaldi et al., 2015). The suspension was centrifuged at 14,000 × *g* at 4°C for 35 min, and the supernatant was stored at −80°C for further analysis. Total protein content was determined using bovine serum albumin as a standard (Bradford, 1976). SOD activity was expressed as unities of SOD per mg of protein.

The activity of superoxide dismutase (SOD) in the spectrophotometer was determined with the method described by Giannopolitis and Ries (1975), in which the activity is measured by the ability of the enzyme to inhibit the photochemical reduction of the compound tetrazolium-nitroblue chloride (NBT). The reaction solution (3 ml) was composed of a 50- mmol L^−1^ Na-phosphate buffer (pH 7.8), 75 μmol L^−1^ NBT, 3 μmol L^−1^ riboflavin, 13 mmol L^−1^ methionine, 0.1 mmol L^−1^ EDTA, and 50 μl of protein extract. The solution was added to glass tubes and illuminated with a 15 -W fluorescent lamp for 5 min. After the period of exposure, the solution was analyzed in a spectrophotometer model Genesis 10S UV-Vis (Thermo Fisher Scientific, Waltham, MA, United States) at 560 nm.

The measurement of H_2_O_2_ and lipid peroxidation content was performed on the same extraction, in which 0.3 g of frozen fine leaf powder (from the second vegetative flush) mass of the leaves was homogenized in 0.1% (w/v) trichloroacetic acid and 4% (w/v) polyvinylpolypyrrolidone (PVPP) centrifuged at 5,590 × *g* for 15 min at 4°C (Alexieva et al., 2001). For H_2_O_2_ content, the supernatant was mixed with a 100-mmol L^−1^ potassium phosphate buffer (pH 7.0), and 1 mol L^−1^ potassium iodide (1:1:4) and incubated at 4°C for 1 h in darkness and after 20 min at 25°C. The absorbance of the samples was measured at 390 nm (Multiskan Go; Thermo Fisher Scientific, Waltham, MA, United States) and calculated using a standard curve with known concentrations of H_2_O_2_. Lipid peroxidation was determined by the presence of malondialdehyde (MDA) according to Heath and Packer (1968). To the supernatant sample, 1 ml of a solution containing 20% (w/v) trichloroacetic acid and 0.5% (w/v) thiobarbituric acid was added and then incubated at 95°C for 30 min followed by quick cooling at 4°C to stop the reaction. The samples were re-centrifuged for 5 min at 12,100 × *g*, and the supernatant was measured at 535 and 600 nm (Multiskan Go; Thermo Fisher Scientific, Waltham, MA, United States). The absorbance of TBA reactive substances that formed was determined at 535 nm. The measurements were corrected for unspecific turbidity by subtracting the absorbance at 600 nm. Using an extinction coefficient of 155 mmol L^−1−1^ cm^−1^, the amount of MDA was calculated.

### Carbon Isotope Composition (δ^13^C)

Approximately 0.01 m of the tips of both branches of the second vegetative flush were dried in an air forced ventilation oven at 65°C for 72 h, ground in a mill grinder (Willy–MSSL-031). Thereafter, a range of *ca*. 2 mg of sample were weighed out in tin capsules. Thereafter, the carbon (^13^C/^12^C) isotope ratio of the samples was analyzed in an Isoprime isotope ratio mass spectrometer (Micromass, Wilmslow, United Kingdom) coupled to an elemental analyzer (Eurovector, Pavia, Italy). Isotopic C-ratio was calculated using the following standard δ notation:


(9) $$\begin{array}{l} \delta^{13}C={\left[\left(\frac{\text{Rsample}}{\text{Rreference}}\right)-1\right]}^{*}1000\left(‰\right) \end{array}$$

where R = ^13^C/^12^C for carbon. The isotope ratios were calibrated against the international standards IAEA CH6 and IAEA CH7. δ^13^C results were referenced against Pee Dee Belemnite (PDB). Precision (the standard deviation of the set of standards analyzed in each batch) was 0.06‰.

### DNA Isolation and CLas Quantification

Deoxyribonucleic acid extraction was performed according to the CTAB method originally described in Murray and Thompson (1980). Briefly, fine roots (*ca*. 2 mm) and the petiole of six fully expanded leaves were collected, cut into small pieces, and placed in 2-ml plastic tubes (200 mg each) containing 5-mm beads and 625 μl of buffer 1 (100 mmol L^−1^ Tris [pH 8], 50 mmol L^−1^ EDTA, and 500 mmol.L^−1^ NaCl). The plant tissue was ground in a homogenizer TissueLyzer II (Qiagen, Germantown, MD, United States) at 30 Hz for 120 s, and then 725 μl of buffer 2 [5% CTAB, 10% Sarcosyl (Sigma-Aldrich, St. Louis, MO, United States), and 10 mmol L^−1^ B-mercaptoethanol] was added to the tube. This was held at 65°C for 30 min and then centrifuged for 5 min at 3,500 rpm. The supernatant was transferred to a new 1.5-ml microtube, extracted with chloroform/isoamyl alcohol 24:1, and precipitated with isopropanol; the total DNA was suspended in 400 μl of 1/10TE + RNase (pancreatic ribonuclease, EC 3.1.27.5). DNA concentration was quantified by spectrophotometry at 260/280 OD ratio using the nanodrop system (Thermo Fisher Scientific, Wilmington, NC, United States). The DNA was standardized to a 100-ng μl^−1^ concentration.

Real time quantitative PCR (q-PCR) was performed with a total volume of 13.5 μl [3 μl of total DNA, 6.75 μl of 2x Maxima® Probe/Rox qPCR Master mix buffer (Fermentas, St. Leon-rot, Germany), 0.4 μmol L^−1^ of each primer, 0.2 μmol L^−1^ of FAM /Iowa Black FQ label probe (Integrated DNA Technology, IDT, Coralville, IA, United States) using an ABI7500 (Applied Biosystems, Foster City, CA, United States) under default cycling conditions, totaling 40 cycles. The primers and probes used were based on the TS elongation fraction (efTs) of *C*Las (Hong et al., 2010). All the reactions used internal control primers (GAPDH gene) according to Boava et al. (2015). The results were expressed as the number of PCR cycles, in which *reporter* emission reaches a value higher than the threshold. The C_T_ values are inversely proportional to the concentration of bacteria in the sample, and it has been shown that C_T_ values lower than 36 are considered *C*Las-positive (infected, +) (Ammar et al., 2011; Coletta-Filho et al., 2014; Canale et al., 2017). Samples with C_T_ values between 33 and 36 were reread to certify the positive diagnosis. For the negative control, tissues of the healthy plants were used, with Milli-Q autoclaved (Merck Group, Darmstadt, Germany) water as the mock sample.

### Leaf Area, Root Volume, and Dry Weights

All the leaves were collected, and their area was measured using a portable area meter (LI-3000C; Li-Cor, Lincoln, NE, United States). Root volume was measured using a 2-L graduated cylinder containing a known volume of water (V1). The roots were then submerged into the water, and the new volume was recorded (V2). Root volume was calculated as the difference between V1 and V2.

The leaves, stems, and roots were dried at 65°C for 72 h and weighed (± 0.001 g) for dry weight determination. Leaf, stem, and root dry weight values were then used to calculate both the biomass allocation (%) and the relation between shoot (leaf dry weight + stem dry weight) and root dry weights (root: shoot ratio).

### Statistical Analysis

A complete randomized design was set up in a split plot scheme to assess the effect of HLB disease condition in citrus and its interaction with water management regime during plant growth. Two levels of HLB [healthy (HLB–) or infected (HLB+)] were the main plot factor, and two levels of management supply [fully irrigated (FI) or deficit-irrigated (DI)] were the subplot factor. Treatment combinations were repeated eight times, totaling 32 experimental units in this study. Dependent variables were tested by ANOVA followed by Student's unpaired *t*-test at 5% probability. Statistical methods were conducted using the software ASSISTAT 7.0 BETA.

## Results

### Leaf Area, Root Volume, and Dry Weights

HLB infection reduced growth. The HLB+ FI plants developed smaller leaf area and lower root volume, root: shoot ratio, and stem, shoot, and root dry weights than the HLB– FI plants (Table 1). These trends were not observed for the HLB+ DI and HLB– DI plants; therefore, no differences were observed between the two treatments for all variables shown in Table 1.

**Table 1:** Leaf area, root volume and leaf, stem shoot and root dry weights of “Valência” orange trees [*Citrus sinensis* (L.) Osbeck] grafted on citrumelo “Swingle” rootstock [*Citrus paradisi* Macfad. x *Poncirus trifoliata* (L.) Raf.] either infected with “*Ca*. Liberibacter asiaticus”.

**Plant biomass component**	**HLB+**		**HLB-**		
	**FI**	**DI**	**FI**	**DI**	**CV (%)**
Leaf dry weight (g)	25.76^aA^	19.17^aB^	29.26^aA^	19.69^aB^	13.81
Stem dry weight (g)	40.69^bA^	36.34^aA^	57.12^aA^	37.44^aB^	8.28
Shoot dry weight (g)	66.45^bA^	55.51^aB^	86.38^aA^	57.13^aB^	13.49
Root dry weight (g)	34.69^bA^	40.68^bA^	62.14^aA^	50.21^aB^	16.41
Root:shoot ratio	0.54^bB^	0.74^aA^	0.72^aB^	0.88^aA^	17.64
Leaf area (cm^−2^)	1,464.53^bA^	1,304.40^aA^	2,101.49^aA^	1,359.06^aB^	17.36
Root volume (dm^3^)	0.08^bA^	0.10^bA^	0.18^aA^	0.13^aB^	19.03

*(+) or healthy (–) and exposed to two water management treatments: fully irrigated (FI) and deficit irrigated (DI). Values are mean ± SE (n = 8). Means followed by the same letter are not significantly different between water management treatments within the same HLB infection conditions (upper case) or between HLB infection conditions within the same water management treatment (lower case) according to unpaired Student's t-test at 5% probability*.

The use of DI reduced leaf and shoot dry weight values of both the HLB+ and the HLB– plants (Table 1). The HLB– DI plants also exhibited lower values of leaf area, root volume, and stem and root dry weights than the HLB– FI plants (Table 1). Nonetheless, no differences between the HLB+ FI and the HLB+ DI plants were observed for leaf area, root volume, and stem and root dry weight (Table 1). Moreover, the HLB+ FI plants exhibited a lower root: shoot ratio than HLB+ DI (Table 1). HLB– FI also showed a reduced root:shoot ratio compared with HLB– DI (Table 1). Phenotype images are shown in Supplementary Figures 1, 2.

### Volumetric Substrate Water Content and Total Water Applied

The HLB+ FI plants required less water to saturate the substrate than the HLB– FI plants throughout the experiment (Supplementary Figure 3A). The HLB+ FI plants consumed an average of 16.6% less water than the HLB– FI plants (Supplementary Figure 3A). Similarly, less water was applied to the HLB+ DI plants in relation to those of HLB– DI (Supplementary Figure 3A).

### Leaf Water Potential

**Figure 2:**
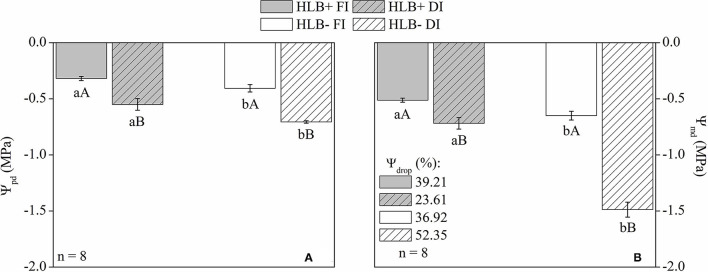
Leaf water potential at predawn [Ψ_*pd*_, **(A)**], midday [Ψ_*md*_, **(B)**], and drop (%) of Ψ_*md*_ in relation to Ψ_*pd*_ [Ψ_*drop*_, **(B)**] of leaves of “Valência” orange trees [*Citrus sinensis* (L.) Osbeck] grafted on citrumelo “Swingle” rootstock [*Citrus paradisi* Macfad. x *Poncirus trifoliata* (L.) Raf.] either infected with “*Ca*. Liberibacter asiaticus” (+) or healthy (–) and exposed to two water management treatments: fully irrigated (FI) and deficit-irrigated (DI). Values are mean ± SE. (*n* = 8). Means followed by the same letter do not differ between water management treatments within the same HLB infection condition (upper case) or between HLB infection conditions within the same water management treatment (lower case) according to unpaired Student's *t*-test at 5% probability.

Both Ψ_*pd*_ and Ψ_*md*_ were higher (less negative) in the HLB+ plants, regardless of water management treatment (Figure 2). On the other hand, DI reduced both Ψ_*pd*_ and Ψ_*md*_, regardless of HLB infection conditions (Figure 2). Compared with the corresponding FI treatment, DI reduced the Ψ_*md*_ of HLB+ and HLB– by 28.7 and 56.3%, respectively (Figure 2B). Moreover, Ψ_*drop*_ was larger in HLB– DI, whereas HLB+ DI presented the lowest values of Ψ_*drop*_ (Figure 2B).

### Leaf Gas Exchanges and δ^13^C

**Figure 3:**
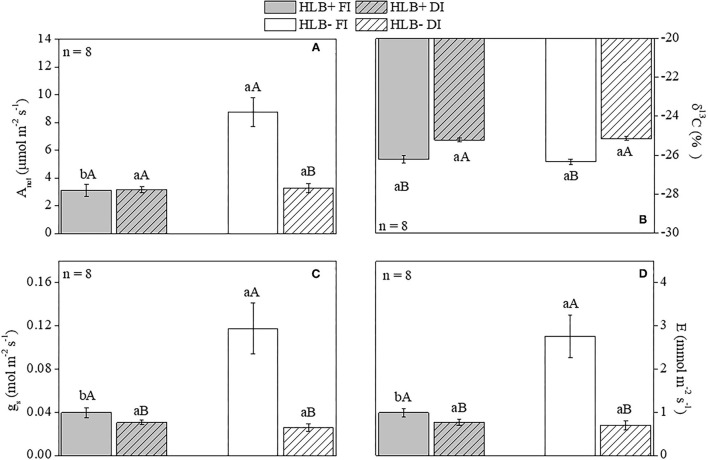
Net photosynthetic rate [*A*_*net*_, **(A)**], carbon isotope composition [δ^13^*C*, **(B)**], stomatal conductance [*g*_*s*_, **(C)**], and transpiration [*E*, **(D)**] of leaves of “Valência” orange trees [*Citrus sinensis* (L.) Osbeck] grafted on citrumelo “Swingle” rootstock [*Citrus paradisi* Macfad. x *Poncirus trifoliata* (L.) Raf.] either infected with “*Ca*. Liberibacter asiaticus” (+) or healthy (–) and exposed to two water management treatments fully irrigated (FI) and deficit-irrigated (DI). Values are mean ± SE (*n* = 8). Means followed by the same letter do not differ between water management treatments within the same HLB infection condition (upper case) or between HLB infection conditions within the same water management treatment (lower case) according to unpaired Student's *t*-test at 5% probability.

Higher *A*_*net*_, *g*_*s*_, and *E* were observed in the HLB– FI plants in relation to both HLB+ FI and HLB– DI (Figures 3A,C,D). Nonetheless, no differences between HLB+ DI and HLB– DI were observed for *A*_*net*_, *g*_*s*_, and *E* (Figures 3A,C,D). Regarding the HLB+ treatments, no differences were observed for *A*_*net*_, whereas HLB+ FI presented higher values for both *g*_*s*_ and *E* in relation to HLB+ DI (Figures 3A,C,D). The DI plants were less depleted in ^13^C (higher δ^13^C, lower discrimination against ^13^C) than the FI plants (higher discrimination against ^13^C) (Figure 3B). HLB infection did not change δ^13^*C*, since no differences were observed between treatments with the same water management treatment (HLB+ FI *x* HLB– FI and HLB+ DI *x* HLB– FI; Figure 3B).

**Figure 4:**
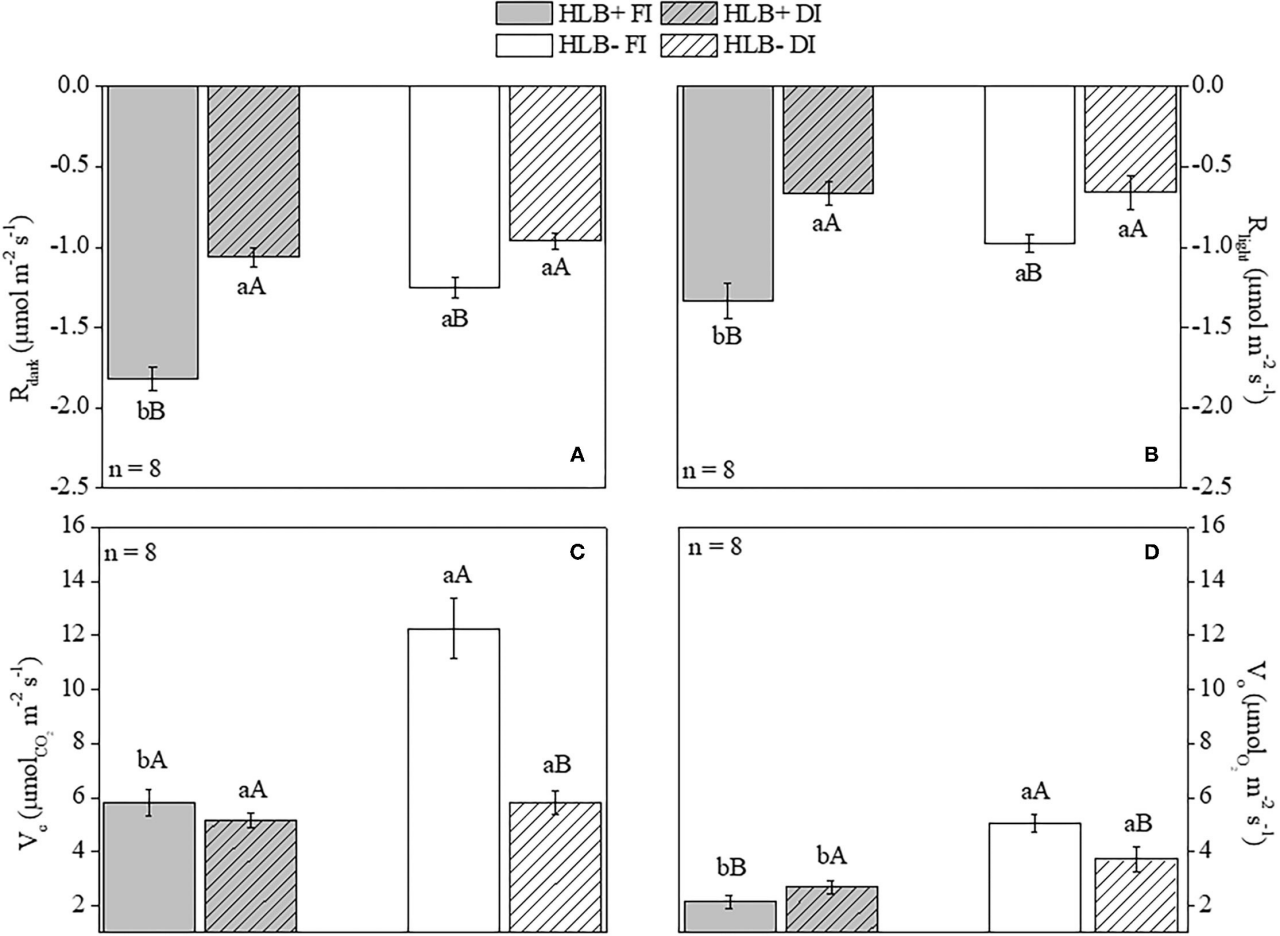
Leaf respiration in the dark [*R*_*dark*_, **(A)**] and in the light [*R*_*light*_, **(B)**], and Rubisco carboxylation [*V*_*c*_, **(C)**] and oxygenation [*V*_*o*_, **(D)**] rates of “Valência” orange trees [*Citrus sinensis* (L.) Osbeck] grafted on citrumelo “Swingle” rootstock [*Citrus paradisi* Macfad. x *Poncirus trifoliata* (L.) Raf.] either infected with “*Ca*. Liberibacter asiaticus” (+) or healthy (–) and exposed to two water management treatments: fully irrigated (FI) and deficit-irrigated (DI). Values are mean ± SE (*n* = 8). Means followed by the same letter do not differ between water management treatments within the same HLB infection conditions (upper case) or between HLB infection conditions within the same water management treatment (lower case) according to unpaired Student's *t*-test at 5% probability.

The HLB+ FI plants had higher values for both *R*_*dark*_ and *R*_*light*_ than for both HLB+ DI and HLB– FI (Figures 4A,B). DI reduced leaf respiration rates in both the healthy (HLB–) and the infected (HLB+) plants (Figures 4A,B). Overall, the FI plants exhibited higher *V*_*c*_ than the DI plants, regardless of infection conditions (Figure 4C). The HLB+ FI plants presented lower *V*_*c*_ and *V*_*o*_ rates than the HLB– FI plants (Figures 4C,D). Although DI reduced *V*_*c*_ in the HLB– plants, this trend was not observed between the HLB+ treatments (Figure 4C).

### Chlorophyll a Fluorescence and JIP-Test Measurements

**Figure 5:**
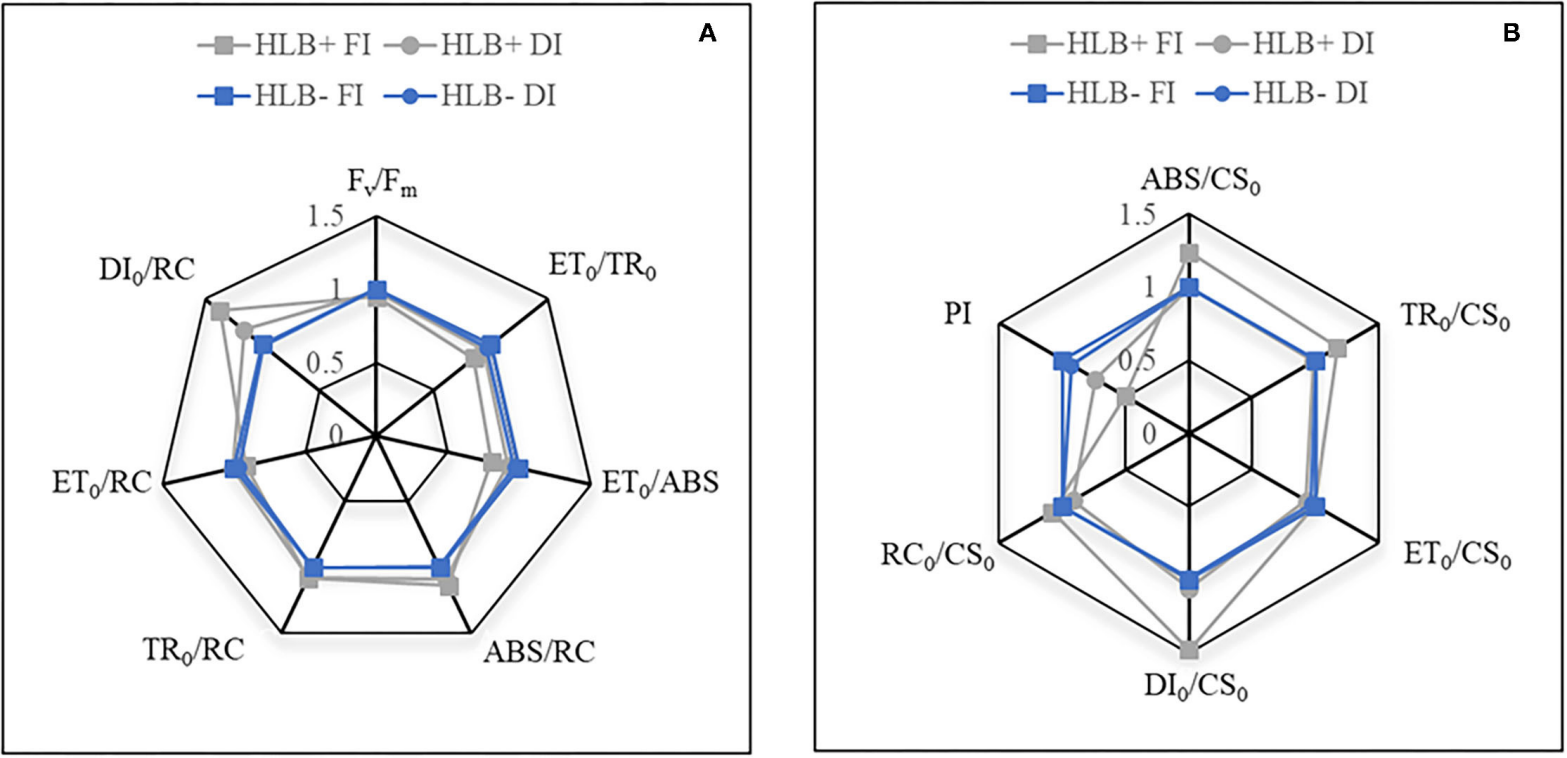
**(A,B)** Relative changes in chlorophyll *a* fluorescence traits of leaves of “Valência” orange trees [*Citrus sinensis* (L.) Osbeck] grafted on citrumelo “Swingle” rootstock [*Citrus paradisi* Macfad. x *Poncirus trifoliata* (L.) Raf.] either infected with “*Ca*. Liberibacter asiaticus” (+) or healthy (–) and exposed to two water management treatments: fully irrigated (FI) and deficit-irrigated (DI). ABS/CS_0_, number of photons absorbed by an excited PSII cross-section; ABS/RC, effective antenna size of an active reaction center; DI_0_/CS_0_, energy dissipation rates per cross-section; DI_0_/RC, effective dissipation of an active reaction center; ET_0_/ABS, quantum yield of electron transport; ET_0_/CS_0_, electron transport in a PSII cross-section; ET_0_/RC, electron transport in an active reaction center; ET_0_/TR_0_, electron transport probability; Fv/Fm, maximum quantum yield of primary photochemistry; PI: photosynthetic index; RC/CS_0_, fraction of active reaction centers per excited cross-section of leaf; TR_0_/CS_0_, maximal trapping rate in a PSII cross-section; TR_0_/RC, maximal trapping rate of PSII.

Chlorophyll *a* fluorescence traits associated with phenomenological fluxes, such as *ABS/CS*_0_ and *TR*_0_*/CS*_0_, were higher in the HLB+ FI plants (Figure 5B). Nonetheless, the HLB+ FI plants also exhibited reduced values for *ET*_0_*/ABS* and *ET*_0_*/TR*_0_ (Figure 5A). Although HLB infection did not deactivate reaction centers (refer to *RC*_0_*/CS*_0_ and *TR*_0_*/CS*_0_; Figure 5B), the HLB+FI plants presented higher energy dissipation rates per RC (*DI*_0_*/RC*) and per CS_0_ (*DI*_0_*/CS*_0_) (Figure 5). Changes in both *DI*_0_*/RC* and *DI*_0_*/CS*_0_in the HLB+ DI plants in relation to the standard (HLB– FI) were lower (Figure 5). Thereby, the HLB+ plants presented reduced values for PI, especially under FI conditions (Figure 5B). Overall, we did not observe differences between HLB– FI and HLB– DI (Figure 5).

### Hydrogen Peroxide, Lipid Peroxidation, and Superoxide Dismutase (SOD) Activity

**Figure 6:**
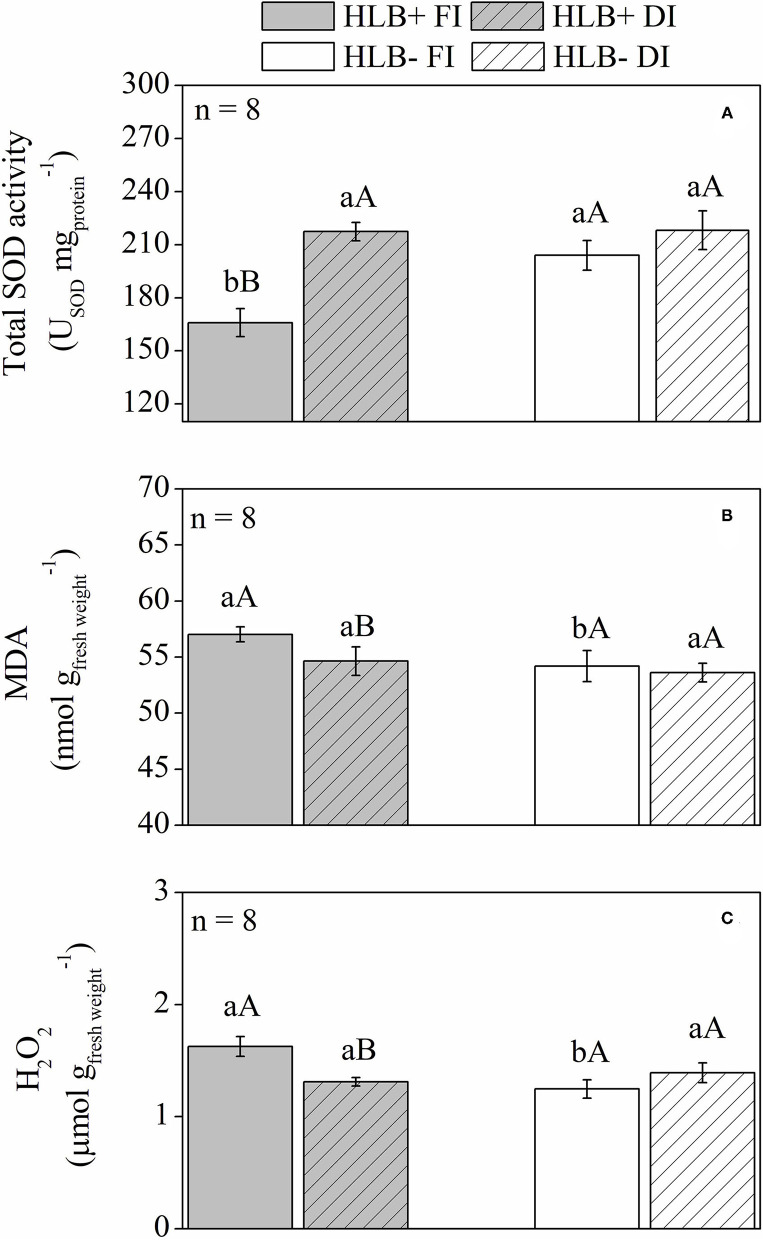
Superoxide dismutase activity [*SOD*, **(A)**], lipid peroxidation [*MDA*, **(B)**], and hydrogen peroxide content [H_2_O_2_, **(C)**] of leaves of “Valência” orange trees [*Citrus sinensis* (L.) Osbeck] grafted on citrumelo “Swingle” rootstock [*Citrus paradisi* Macfad. x *Poncirus trifoliata* (L.) Raf.] either infected with “*Ca*. Liberibacter asiaticus” (+) or healthy (–) and exposed to two water management treatments: fully irrigated (FI) and deficit-irrigated (DI). Values are mean ± SE (*n* = 8). Means followed by the same letter do not differ between water management treatments within the same HLB infection conditions (upper case) or between HLB infection conditions within the same water management treatment (lower case) according to unpaired Student's *t*-test at 5% probability.

The HLB+ FI plants presented lower SOD activity, associated with higher contents of MDA and H_2_O_2_ in relation to both HLB+ DI and HLB– FI (Figure 6). No differences were observed between HLB+ DI and HLB– DI for MDA and H_2_O_2_ contents and SOD activity (Figure 6). The same was observed between HLB– FI and HLB– DI (Figure 6).

### Quantification of Starch and Soluble Sugars in Leaves

**Figure 7:**
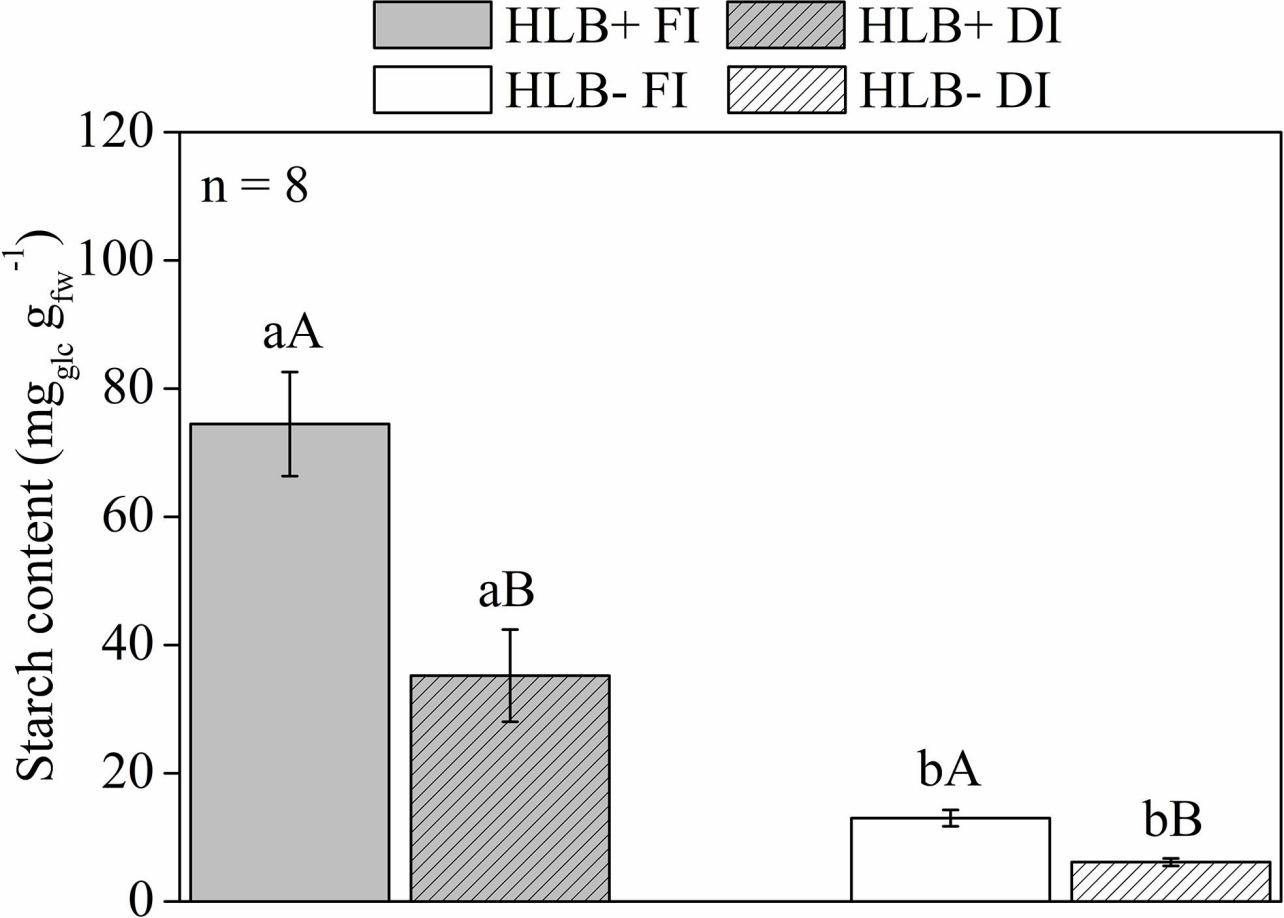
Starch content of leaves of “Valência” orange trees [*Citrus sinensis* (L.) Osbeck] grafted on citrumelo “Swingle” rootstock [*Citrus paradisi* Macfad. x *Poncirus trifoliata* (L.) Raf.] either infected with “*Ca*. Liberibacter asiaticus” (+) or healthy (–) and exposed to two water management treatments: fully irrigated (FI) and deficit-irrigated (DI). Values are mean ± SE (*n* = 8). Means followed by the same letter do not differ between water management treatments within the same HLB infection conditions (upper case) or between HLB infection conditions within the same water management treatment (lower case) according to unpaired Student's *t*-test at 5% probability.

**Figure 8:**
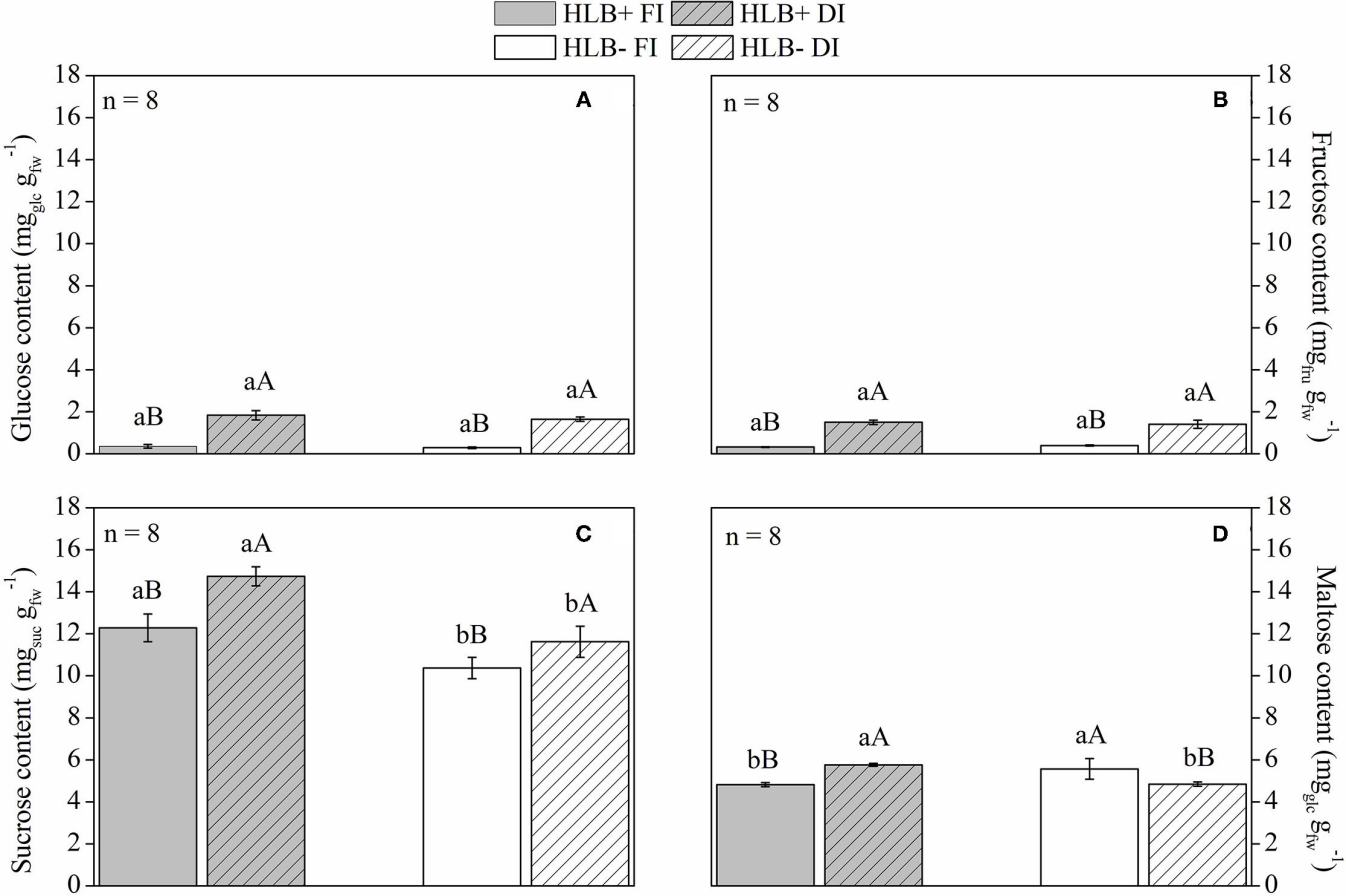
**(A)** Glucose, **(B)** fructose, **(C)** sucrose, and **(D)** maltose **(D)** content of leaves on “Valência” orange trees [*Citrus sinensis* (L.) Osbeck] grafted on citrumelo “Swingle” rootstock [*Citrus paradisi* Macfad. x *Poncirus trifoliata* (L.) Raf.] either infected with “*Ca*. Liberibacter asiaticus” (+) or healthy (–) and exposed to two water management treatments: fully irrigated (FI) and deficit-irrigated (DI). Values are mean ± SE. (*n* = 8). Means followed by the same letter do not differ between water management treatments within the same HLB infection conditions (upper case) or between HLB infection conditions within the same water management treatment (lower case) according to unpaired Student's *t*-test at 5% probability.

The HLB+ plants exhibited higher leaf starch content than the corresponding HLB– plants (Figure 7). Both DI treatments reduced leaf starch content in relation to the corresponding FI treatments (Figure 7). Thereby, both DI treatments had higher glucose, fructose, and sucrose contents than the respective FI treatments (Figures 8A–C). HLB+ DI presented higher maltose content than HLB+ FI, whereas the opposite was observed between the HLB– DI and the HLB– FI plants (Figure 8D). Although no differences were observed for glucose and fructose, we measured higher sucrose and lower maltose contents in HLB+ FI in relation to HLB– FI (Figure 8). Finally, the HLB+ DI plants exhibited higher sucrose and maltose contents than the HLB– DI plants (Figures 8C,D).

### CLas Quantification

**Figure 9:**
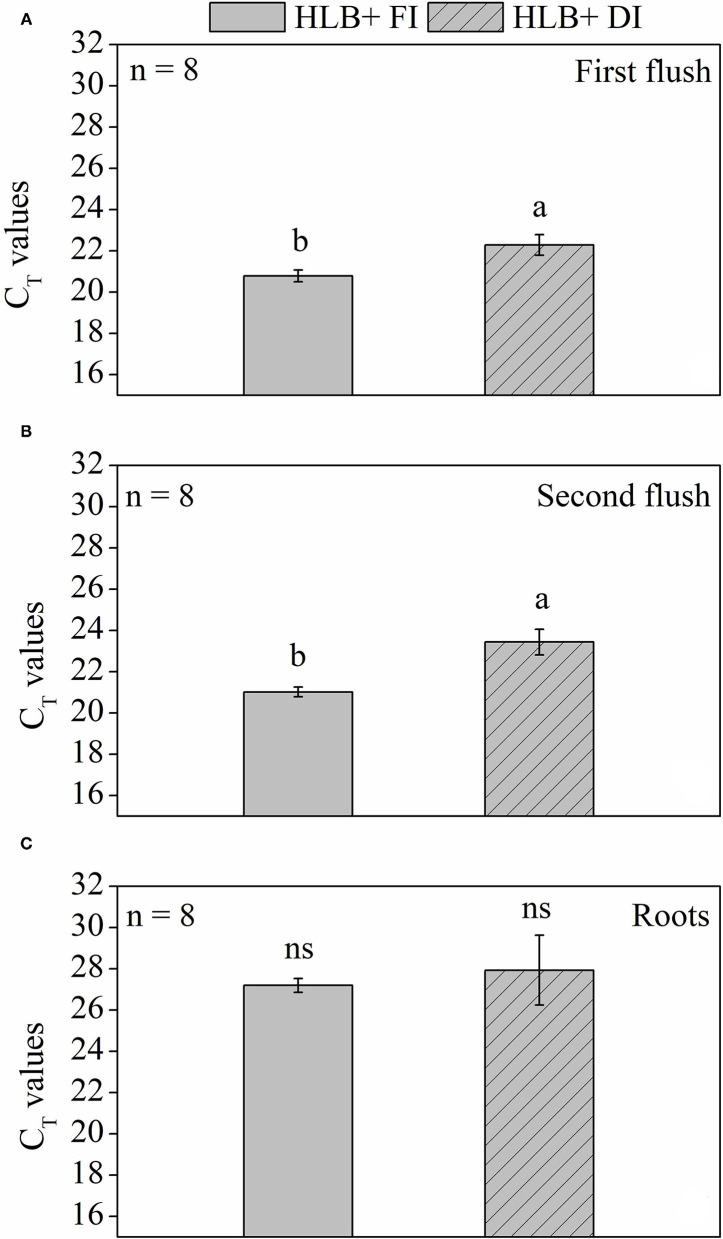
Cycle threshold (C_T_) values of leaves from the **(A)** first and **(B)** second vegetative flush and fine roots **(C)** of “Valência” orange trees [*Citrus sinensis* (L.) Osbeck] grafted on citrumelo “Swingle” rootstock [*Citrus paradisi* Macfad. x *Poncirus trifoliata* (L.) Raf.] infected with “*Ca*. Liberibacter asiaticus” and exposed to two water management treatments: fully irrigated (FI) and deficit-irrigated (DI) 225 days after transferring the plants to the 12-L pots. Means followed by the same letter do not differ according to unpaired Student's *t*-test at 5% probability. *ns* indicates no statistical difference. Values are mean ± SE (*n* = 8).

At 225 days after transferring plants to the 12 L pots, the *CLas* bacterial titer in the roots, estimated by C_T_ values, was not affected by water management treatments and was lower than the values observed in the leaves (Figure 9). Higher C_T_ values (i.e., lower bacteria content) were observed in HLB+ DI in both first and second vegetative flushes (Figures 9A,B). Nonetheless, C_T_ values were lower than 24 in both HLB+ FI and HLB+ DI treatments, which indicates high pathogen concentration.

## Discussion

### Water Relationship of HLB-Infected Plants Exposed to Different Water Management Treatments

We observed that the HLB+ FI plants grew less and consumed a reduced volume of water despite presenting higher volumetric substrate moisture in relation to the HLB– FI plants (Supplementary Figure 3). Such results indicate that the HLB+FI plants presented limited water uptake capacity. It has been reported that limitations to water movement in HLB-infected plants are associated to root growth impairment (Graham et al., 2013; Kadyampakeni et al., 2014; Hamido et al., 2016), as observed in this study (Table 1). Nonetheless, we demonstrated that physiological traits other than root growth are also involved in this process. Indeed, water transport in the HLB+ FI plants was limited not only by reduced growth but also by lower *g*_*s*_ and *E* (Figures 3C,D). Leaf area was also smaller in the HLB+ FI than in the HLB– FI plants (Table 1), which reduces total shoot transpiration (Galvez et al., 2011; Pedroso et al., 2014). Therefore, stomatal effects (Figures 3C,D), in combination with a reduced root system and leaf area (Table 1), decreased water uptake in the HLB+ FI plants (Supplementary Figure 3A). These results, associated with higher Ψ_*pd*_ and Ψ_*md*_ in HLB+ FI (Figure 2), strongly support that the HLB+ plants exhibit reduced hydraulic conductance compared with the HLB– plants (Ribeiro et al., 2009). Thus, substrate water content reduces slowly in plants with limited hydraulic conductance (Pérez-Pérez et al., 2009), as observed in the HLB+ FI treatment (Table 1; Figures 2, 3; and Supplementary Figure 3A).

Stomatal closure is likely a direct effect of the interplay between microorganisms and plant compounds secreted during the plant-pathogen interaction, but limited to epiphytic species and related to high concentrations of bacteria (Gudesblat et al., 2009). Considering this is not the case of *C*Las, a systemic pathogen directly introduced in the plant by the vector, we argue that the variations in stomatal conductance observed in our study occurred in parallel to changes in the hydraulic conductance in infected plants. Similarly, *g*_*s*_ decreased in citrus during the infection with xylem-limited bacteria *Xylella fastidiosa*, which caused xylem occlusion and restricted water flows to the leaves (Habermann et al., 2003).

Plants can undergo stress when water uptake of roots fails to compensate evapotranspiration rates (Fereres and Soriano, 2007), so we would expect that HLB– FI had an improved water status compared with HLB+ FI (Etxeberria et al., 2009; Johnson et al., 2014; Kumar et al., 2018). However, lower values of leaf area (Table 1), *E*, and *g*_*s*_ (Figures 3C,D) also reduced water loss in the HLB+ FI plants, mitigating the damages to water status in this treatment, as supported by the higher values of both Ψ_*pd*_ and Ψ_*md*_ in relation the HLB– FI plants (Figure 2). Such reductions in *g*_*s*_ and *E* were proportional to the decrease in *A*_*net*_ (Figure 3A). Therefore, we did not observe significant changes in δ^13^C (Figure 3B). There is a good relationship among δ^13^C, *A*_*net*_, and *g*_*s*_ (Chaves et al., 2007), so δ^13^C has been used to determine the water use efficiency of plants grown under different environmental conditions (Farquhar and Richards, 1984; Busch et al., 2020). Considering that δ^13^C integrates photosynthetic activity and water relationships (Aranjuelo et al., 2009), throughout the period over which the tissue was synthesized (Salazar-Parra et al., 2015), we can assume that HLB infection did not affect water use efficiency (Figure 3D). In addition, respiration can contribute to the depletion of δ^13^C (Badeck et al., 2005; Chaves et al., 2007; Busch et al., 2020). Thus, despite the low *g*_*s*_ of HLB+ FI (Figure 3C), δ^13^C did not vary between FI treatments given the increase in both *R*_*light*_ and *R*_*dark*_ caused by HLB infection (Figures 4A,B).

Both DI treatments exhibited lower (more negative) Ψ_*pd*_ and Ψ_*md*_ than the respective FI treatments (Figure 2). In addition, the DI plants invested more energy in root than in shoot growth, since a higher root:shoot ratio was observed in relation to the respective FI treatment (Table 1). Higher investments in root growth in response to reduced water availability (Lei et al., 2006) can improve water uptake (Galvez et al., 2011). The negative effects of DI on plant water status were not intensified by HLB+ infection; but on the contrary, both Ψ_*pd*_ and Ψ_*md*_ were higher in the HLB+ DI than in the HLB– DI plants (Figure 2). Ψ_*drop*_ was larger in HLB– DI, whereas HLB+ DI had the lowest values of Ψ_*drop*_ (Figure 2B). Both DI treatments also had lower discrimination against δ^13^C (Figure 3B), which is a positive response of the DI plants since it represents improved water use efficiency (Pérez-Pérez et al., 2009; Panigrahi et al., 2014; Romero-Conde et al., 2014). Altogether, our results show that the irrigation of HLB-infected plants need to be accurately designed to aid the best irrigation management to HLB-infected trees, and that future research is required to determine the best irrigation level for HLB+ trees while also considering its effects on fruit quality and yield.

### DI Alleviates the Negative Effects of HLB

The HLB+ DI plants displayed higher C_T_ values in both the first and second vegetative flushes of leaf growth (Figures 9A,B). Considering that the first vegetative flush of growth had tested positive for HLB before the initiation of water management treatments, DI contributed to alleviate *C*Las population increase in relation to HLB+ FI. This response is likely associated with the high leaf respiration rates observed in the HLB+ FI plants (Figures 4A,B). Citrate, a subproduct of the TCA cycle, has been presumed to be a main source of energy for *C*Las (Cruz-Munoz et al., 2018), and high *R* values could accelerate *C*Las growth. Nonetheless, conditions that decrease both *R*_*light*_ and *R*_*dark*_, such as deficit irrigation (Figures 4A,B), can contribute to hindering bacterial growth (Figures 9A,B). However, the HLB+ DI plants still presented high concentrations of bacteria, and no effects were observed in the roots (Figure 9).

A 9% reduction in *C*Las population was associated with less starch content in HLB+ DI in relation to HLB+ FI (Figure 7), even though the process associated with starch synthesis/breakdown in the HLB-infected trees has not been fully elucidated (Etxeberria et al., 2009; Gibon et al., 2009; Fan et al., 2010). Our results also provide evidence that both starch synthesis and breakdown are impaired in the HLB+ plants, but were partially alleviated by DI (Figure 7).

Higher starch content in HLB+ FI (Figure 7) was observed despite the highest *R* values (Figures 4A,B) and lower *A*_*net*_ and *V*_*c*_ in relation to HLB– FI (Figures 3A, 4C). *R* impacts plant carbon balance since a variable proportion of CO_2_ assimilated by photosynthesis is released back to the atmosphere through respiration (Ayub et al., 2014). Changes in this process can result in substantial variation in C availability for plant maintenance and growth (Flexas et al., 2010; Griffin and Heskel, 2013). Taking into account that less C was available in HLB+ FI in relation to both HLB+ DI and HLB– FI (Figures 3A, 4A–C), most of the C was converted into starch in the HLB+ FI plants (Figure 7).

The accumulation of lower biomass in HLB+ FI in relation to HLB– FI would suggest that more starch was accumulated in HLB+ FI since less carbon was used to promote plant growth (Table 1; Figure 7). Although it can contribute to starch accumulation given the damages HLB causes on the transport of photosynthates to growing tissues (Etxeberria et al., 2009; Graham et al., 2013, Johnson et al., 2014; Kadyampakeni et al., 2014; Hamido et al., 2016), overall, biomass production did not differ between both HLB+ treatments (Table 1), so starch synthesis was upregulated in the HLB+ FI plants (Figure 7).

The HLB+ FI plants had lower maltose in leaves in relation to HLB– FI (Figure 8D), and the HLB+ DI plants exhibited higher sucrose and maltose content compared with the HLB– DI plants (Figures 8C,D). Since maltose is the main product of starch degradation (Zeeman et al., 2004; Fan et al., 2010; Aritua et al., 2013), it is clear that starch breakdown was also downregulated by HLB, which was alleviated by DI. Indeed, the HLB+ FI plants exhibited lower glucose, fructose, sucrose, and maltose content compared with the HLB+ DI plants (Figure 8).

Carbohydrate reserves decrease in storage organs, as plants are exposed to reduced water availability (Meltcafe et al., 2010). Because of reduced photosynthetic capacity under DI conditions, soluble sugars from starch degradation can be used to maintain plant metabolic activity (Dickson and Isebrands, 1991), so increases in soluble sugar contents were observed (Chaves et al., 2009; Pedroso et al., 2014; Puglisi et al., 2019), as shown in HLB+ DI (Figures 7, 8).

Nonstructural sugars, such as sucrose, are important signaling molecules and play important roles in adaptive mechanisms, such as sucrose induction of stress defense under reduced water availability conditions (Hanson and Smeekens, 2009; Ramel et al., 2009; Pinheiro and Chaves, 2011). The increase in glucose, fructose, sucrose, and maltose content in the HLB+ DI plants (Figure 8) could have triggered a series of protection events against impairments in the physiology of the HLB+ plants (Ramel et al., 2009; Pinheiro and Chaves, 2011). Although the HLB+ FI plants had lower soluble sugar contents in leaves (Figure 8), they also exhibited a reduced capacity to move electrons into the electron transport chain beyond QA^−^, since most of the absorbed (ABS/CS_0_) and trapped (TR/CS_0_) energy was dissipated (DI_0_/CS_0_ and DI_0_/RC) (Figure 5). Even though this process was not associated with the deactivation of reaction centers, it was followed by reduced values of PI (Figure 5B).

This apparent blockage of electron transfer between the acceptors (Shu et al., 2012) may have damaged the initial stage of photosynthetic activity of a non-impaired RC complex (Strauss et al., 2006; Zlatev, 2009) in the HLB+ FI plants (Figure 5). Significant reductions of proteins associated with photosystem II stability have been reported in HLB-infected plants (Nwugo et al., 2013). Nonetheless, such changes were not observed in HLB+ DI plants (higher soluble sugar contents, Figure 8), such that trends in chlorophyll *a* fluorescence traits in the HLB+ DI plants were similar to those in the HLB– plants (Figure 5). Thus, our results strongly indicate that the photochemistry of citrus trees is resistant to DI (Pedroso et al., 2014; Romero-Conde et al., 2014). In addition, DI activated specific ROS-scavenging systems triggered by higher soluble sugar contents (Figure 8), which reduced oxidative damages and photoinhibition (Ramel et al., 2009; Pinheiro and Chaves, 2011) caused by HLB+.

The increase of photorespiration (*V*_*o*_) in HLB+ DI over HLB+ FI (Figure 4D), supports an avoidance mechanism against oxidative damages and photoinhibition (Hochberg et al., 2013). The limitation of CO_2_ assimilation rates (*A*_*net*_, Figure 3A), accompanied by an increase in the activity of photorespiration (Figure 4D) as another sink for the absorbed energy, reduced the decline in electron transport (Harbinson et al., 1990; Wingler et al., 1999; Pinheiro and Chaves, 2011) for the HLB+ DI plants (ET_0_/ABS, ET_0_/TR_0_, ET_0_/RC; Figure 5). Such trends in *V*_*o*_ were followed by higher values of SOD activity in HLB+ DI compared with HLB+ FI (Figure 6A).

Since the activity of SOD metalloenzymes is responsible for alleviating oxidative stresses caused by ROS (Alscher et al., 2002; Foyer and Noctor, 2009), we also observed lower MDA and H_2_O_2_ contents in the HLB+ DI leaves in relation to the HLB+ FI plants (Figures 6B,C). Higher MDA values in HLB+FI in relation to HLB+DI plants (Figure 6B) is another indication that the ROS scavenging system was less efficient in alleviating oxidative stress (Dourado et al., 2014; Capaldi et al., 2015) caused by the disease. Although HLB caused a series of oxidative stresses in the plants (Figures 5, 6), the use of DI partially alleviated such damages.

Although HLB infection reduced growth, *A*_*net*_, and *g*_*s*_ (Figures 3A,C), we observed that DI did not aggravate these negative effects, since there were no differences between the *A*_*net*_ of HLB+ FI and HLB+ DI (Figure 3A). Therefore, leaf area, root volume, and stem and root dry weights did not vary between both HLB+ treatments (Table 1). It is important to note that the higher carboxylation rates of HLB+ FI compared with HLB+ DI (*V*_*c*_; Figure 4C), did not improve *A*_*net*_ (Figure 3A), so growth was not substantially modified (Table 1). Much of the carbon assimilated by HLB+ FI was likely consumed by *R* (Figures 4A,B). Nonetheless, the lower *V*_*c*_ and *V*_*o*_ values in the HLB+ plants (Figures 4C,D) are a strong indication of reduced Rubisco or total activity (Aranjuelo et al., 2005; Pinheiro and Chaves, 2011), and are likely due to chloroplast disruption caused by starch accumulation (Stetler et al., 2009; Fan et al., 2010), especially in the HLB+ FI plants (Figure 7).

## Conclusions

Given the importance of HLB, which affects citrus and causes significant losses in fruit yield and quality of commercial orchards, our research characterized biochemical and physiological traits of trees at the plant-pathogen-environment level looking for the joint effect of disease and deficit irrigation. Despite the fact that the disease limits root growth by (i) reducing leaf area, *g*_*s*_, *E* contribute to restricting water uptake in HLB+ compared with healthy ones; (ii) tree water status is likely not negatively affected in HLB+ regardless of water supply compared with the corresponding HLB– treatment. We also demonstrated that (iii) HLB increases leaf respiration rates and starch synthesis, downregulates starch breakdown, blocks electron transferring among acceptors in the electron transport chain of photosynthesis, increases oxidative stress, reduces CO_2_ assimilation, and damages both carboxylation and oxygenation rates of Rubisco; (iv) however, conditions that reduce both leaf respiration and accumulation of starch by increasing soluble sugar contents in leaves, as observed in the HLB+ plants with the mild stress imposed by DI, can reduce bacterial growth (revealed by C_T_ values) and trigger a series of protective metabolic measures against further impairments in the physiology and biochemistry. Such results provide insights into HLB-induced responses in infected citrus trees that can guide future studies to screen for genetic tolerance to HLB and improve management strategies in field orchards.

## Data Availability Statement

The original contributions presented in the study are included in the article/Supplementary Material, further inquiries can be directed to the corresponding author/s.

## Author Contributions

JS conceived the original screening and research plans, performed gas exchange leaf water potential measurements, extracted DNA samples, contributed to carbohydrate, H_2_O_2_, MDA, SOD analysis, analyzed and interpreted the data, and wrote the manuscript. RB supervised the experiment, interpreted the data, and reviewed the manuscript. JL was responsible for tree growth management and weekly SPAD readings and helped with protein and sugar extraction. BS extracted and read sugars, H_2_O_2_, MDA, protein contents and SOD activity, and performed destructive analysis for biomass production. HC-F performed qPCR analysis, interpreted the data, and revised the manuscript. DM conceived the project, supervised the experiment, interpreted the data, and reviewed the manuscript. All authors contributed to the article and approved the submitted version.

## Funding

This work was supported by São Paulo Research Foundation (FAPESP, Grants #2015/13572-8 and #2018/14893-0).

## Conflict of Interest

The authors declare that the research was conducted in the absence of any commercial or financial relationships that could be construed as a potential conflict of interest.

## Publisher's Note

All claims expressed in this article are solely those of the authors and do not necessarily represent those of their affiliated organizations, or those of the publisher, the editors and the reviewers. Any product that may be evaluated in this article, or claim that may be made by its manufacturer, is not guaranteed or endorsed by the publisher.

## Abbreviations

(+): plants infected with “*Candidatus* Liberibacter asiaticus”
(–): plants not infected with “*Ca*. Liberibacter asiaticus”
Γ^*^: CO_2_ compensation point in the absence of respiration
Ψ: leaf water potential
Ψ_*drop*_: drop (%) of leaf water potential at midday in relation to leaf water potential at predawn
Ψ_*md*_: leaf water potential at midday
Ψ_*pd*_: leaf water potential at predawn
δ^13^*C*: carbon isotopic composition
*ABS/CS*_0_: number of photons absorbed by an excited PSII cross-section
*ABS/RC*: effective antenna size of an active reaction center
*A*_*net*_: net photosynthetic rate
*C*_*i*_: intercellular CO_2_ concentrations
*C*Las: *Ca*. Liberibacter asiaticus
*C*_*T*_: cycle threshold values
*DI*: deficit-irrigated plants
DI_0_/RC: effective dissipation of an active reaction center
*E*: transpiration rate
*ET*_0_ */ABS*: quantum yield of electron transport
*ET*_0_ */CS*_0_: electron transport in a PSII cross-section
*ET*_0_ */RC*: electron transport in an active reaction center
*ET*_0_ */TR*_0_: electron transport probability
*FI*: full-irrigated plants
F_v_/F_m_: maximum quantum yield of primary photochemistry; *g*_*s*_—stomatal conductance
HLB: huanglongbing
*J*: photosynthetic electron transport
MDA: malondialdehyde
PI: performance index
PSII: photosystem II
*R*: leaf respiration
*RC*: reaction center
*RC/CS*_0_: fraction of active reaction centers per excited cross-section of leaf
*R*_*dark*_: rate of leaf respiration in the dark
*R*_*light*_: rate of leaf respiration in the light
*ROS*: reactive oxygen species
Rubisco: ribulose-1,5-bisfosfato carboxylase oxygenase
qPCR: real-time quantitative PCR
PPFD: photosynthetic photon flux density
SOD: superoxide dismutase
TCA cycle: tricarboxylic acid cycle
*V*_*c*_: rates of carboxilation of Rubisco
*V*_*o*_: rates of oxygenation of Rubisco
*V*_*DI*_: volume of water required for deficit-irrigated plants
*V*_*F*__I_: average volume of water applied to full-irrigated plants
*TR*_0_ */CS*_0_: maximal trapping rate in a PSII cross-section
*TR*_0_ */RC*: maximal trapping rate of PSII.

## References

[B1] Alexieva V. Sergiev I. Mapelli E. Karanov E. (2001). The effect of drought and ultraviolet radiation on growth and trees markers in pea and wheat. Plant Cell Environ. 24, 1337–1344. 10.1046/j.1365-3040.2001.00778.x

[B2] Alscher R.G. Erturk N. Heath L.S. (2002). Role of superoxide dismutases (SODs) in controlling oxidative stress in plants. J. Expt. Bot. 53, 1331–1341. 10.1093/jexbot/53.372.1331 11997379

[B3] Ammar E-D. Shatters R.G. Jr, Lynch, C. Hall D.G. (2011). Detection and relative titer of ‘*Candidatus*Liberibacter asiaticus' in the salivary glands and alimentary canal of *Diaphorinacitri* (Hemiptera: Psyllidae) vector of citrus huanglongbing disease. Ann.Entomol. Soc. Am. 104, 526–533. 10.1603/AN10134

[B4] Aranjuelo I. Cabrera-Bosquet L. Mottaleb S.A. Araus J.L. Nogues S. (2009). ^13^C/^12^C isotope labeling to study carbon partitioning and dark respiration in cereals subjected to water stress. Rapid Commun. Mass Spectrom. 23, 2819–2828. 10.1002/rcm.4193 19653200

[B5] Aranjuelo I. Pérez P. Hernández L. Irigoyen J.J. Zita G. Martínez-Carrasco R.. (2005). The response of nodulated alfalfa to water supply, temperature and elevated CO_2_: photosynthetic down-regulation. *Physiol*. Plantarum. 123, 348–358. 10.1111/j.1399-3054.2005.00459.x

[B6] Aritua V. Achor D. Gmitter F.G. Albrigo G. Wang N. (2013). Transcriptional and microscopic analyses of citrus stem and root responses to *Candidatus*Liberibacter asiaticus infection. PLoS ONE 8, e73742. 10.1371/journal.pone.0073742 24058486 PMC3772824

[B7] Ayub G. Zaragoza-Castells J. Griffin K.L. Atkin O.K. (2014). Leaf respiration in darkness and in the light under pre-industrial: current and elevated atmospheric CO_2_ concentrations. Plant Sci. 226, 120–130. 10.1016/j.plantsci.2014.05.001 25113457

[B8] Badeck F. Tcherkez G. Nogués S. Piel C. andGhashghaie J. (2005). Post-photosynthetic fractionation of stable carbon isotopes between plant organs – a widespread phenome- non. Rapid Commun. Mass Spectrom. 19, 1381–1391. 10.1002/rcm.1912 15880634

[B9] Bernacchi C.J. Singsaas E.L. Pimentel C. Portis A.R. Long S.P. (2001). Improved temperature response functions for models of Rubisco-limited photosynthesis. Plant Cell. Environ. 24, 253–259. 10.1111/j.1365-3040.2001.00668.x

[B10] Boava L.P. Sagawa C.H.D. Cristofani-Yaly M. Machado M.A. (2015). Incidence of ‘*Candidatus*Liberibacter asiaticus' infected plants among Citrandarins as rootstock and scion under field conditions. Phytopathol. 105, 518–524. 10.1094/PHYTO-08-14-0211-R 25423067

[B11] Bradford M.M. (1976). A rapid and sensitive method for the quantitation of microgram quantities of protein using the principles of protein dye-binding. Anal. Biochem. 72, 248–254. 10.1016/0003-2697(76)90527-3 942051

[B12] Brooks A. Farquhar G.D. (1985). Effect of temperature on the CO_2_/O_2_ specificity of ribulose-1, 5-biphosphate carboxylase/ oxygenase and the rate of respiration in the light. Estimates from gas exchange measurements on spinach. Planta. 165, 397–406. 10.1007/BF00392238 24241146

[B13] Busch F.A. Holloway-Phillipsm M. Stuart-Williams H. Farquhar D.G. (2020). Revisiting carbon isotope discrimination in C_3_ plants shows respiration rules when photosynthesis is low. Nat. Plants. 6, 245–258. 10.1038/s41477-020-0606-6 32170287

[B14] Canale M.C. Tomaseto A.F. Haddad M.L. Coletta-Filho H.D. Lopes J.R.S. (2017). Latency and persistence of ‘*Candidatus*Liberibacter asiaticus' in its psyllid vector, *Diaphorinacitri* (Hemiptera: Liviidae). Phytopathol. 107, 1264–1272. 10.1094/PHYTO-02-16-0088-R 27841960

[B15] Capaldi F.R. Gratão P.L. Reis A.R. Lima L.W. Azevedo R.A. (2015). Sulfur metabolism and stress defense responses in plants. *Trop*. Plant Biol. 8, 60–73. 10.1007/s12042-015-9152-1

[B16] Chai Q. Gan Y. Zhao C. Xu H-L. Waskom R.M. Niu Y.. (2016). Regulated deficit irrigation for crop production under drought stress: a review. Agron. Sustain. Dev. 36: 3. 10.1007/s13593-015-0338-6

[B17] Chaves M.M. Flexas J. Pinheiro C. (2009). Photosynthesis under drought and salt stress: regulation mechanisms from whole plant to cell. Ann. Bot. 103, 551–560 10.1093/aob/mcn125 18662937 PMC2707345

[B18] Chaves M.M. Santos T.P. Souza C.R. Ortuño M.F. Rodrigues M.L. Lopes C.M.. (2007). Deficit irrigation in grapevine improves water-use-efficiency without controlling vigour and production quality. Ann. Appl. Biol. 150, 237–252. 10.1111/j.1744-7348.2006.00123.x

[B19] Cimò G. Bianco R.L. Gonzalez P. Bandaranayake W. Etxeberria E. andSyvertsen J.P. (2013). Carbohydrate and nutritional responses to stem girdling and drought stress with respect to understanding symptoms of Huanglongbing in citrus. Hort Sci. 48, 920–928. 10.21273/HORTSCI.48.7.920

[B20] Coletta-Filho H.D. Daugherty M.P. Ferreira C. Lopes J.R.S. (2014). Temporal progression of “*Candidatus* Liberibacter asiaticus” infection in citrus and acquisition efficiency by *Diaphorinacitri*. Phytopathol. 104, 416–421. 10.1094/PHYTO-06-13-0157-R 24620723

[B21] Cousins A.B. Ghannoum O. von Caemmerer S. Badger M.R. (2010). Simultaneous determination of Rubisco carboxylase and oxygenase kinetic parameters in *Triticum aestivum* and *Zea mays* using membrane inlet mass spectrometry. Plant Cell Environ. 33, 444–452. 10.1111/j.1365-3040.2009.02095.x 20002330

[B22] Cruz-Munoz M. Petrone J.R. Cohn A.R. Munoz-Beristain A. Killiny N. Drew J.C.. (2018). Development of chemically defined media reveals citrate as preferred carbon source for Liberibacter growth. Front. Microbiol.ogy 9, 668. 10.3389/fmicb.2018.00668 29675013 PMC5895721

[B23] Delatte T. Umhang M. Trevisan M. Eicke S. Thorneycroft D. Smith S.M.. (2006). Evidence for distinct mechanisms of starch granule breakdown in plants. J. Biol. Chem. 281, 12050–12059. 10.1074/jbc.M513661200 16495218

[B24] Dickson R.E. Isebrands J.G. (1991). “Leaves as regulators of stress response” in Response of Plants to Pultiple Stresse, eds Mooney H.A. Winner W.E. Pell E. J. (San Diego: Academic Press) p. 4–34.

[B25] Dourado M. N. Souza L. A. Martins P. F. Peters L. P. Piotto F. A. Azevedo R. A. (2014). Burkholderia sp. SCMS54 triggers a global stress defense in tomato enhancing cadmium tolerance. Water Air Soil Poll. 225, 1–16. 10.1007/s11270-014-2159-7

[B26] Etxeberria E. Gonzalez P. Achor D. Albrigo G. (2009). Anatomical distribution of abnormally high levels of starch in HLB-affected Valencia orange trees. Physiol. Mol. Plant Pathol. 74, 76–83. 10.1016/j.pmpp.2009.09.004

[B27] Etxeberria E. Narciso C. (2012). Phloem anatomy of citrus trees: healthy vs. greening-affected. Proc. Fla. Sta.Hortic. Soc. 125, 67–70. Available online at: https://crec.ifas.ufl.edu/media/crecifasufledu/faculty/etxeberria/2012-FSHS.pdf

[B28] Fan J. Chen C. Brlansky R.H. Gmitter J.R.F.G. Li Z.G. (2010). Changes in carbohydrate metabolism in *Citrus sinensis* infected with ‘*Candidatus*Liberibacter asiaticus'. Plant Pathol. 59, 1037–1043. 10.1111/j.1365-3059.2010.02328.x29089035

[B29] Farquhar G.D. Richards R.A. (1984). Isotopic composition of plant carbon correlates with water-use efficiency of wheat genotypes. *Funct*. Plant Biol. 11, 539–552. 10.1071/PP9840539

[B30] Farquhar G.D. von Caemmerer S. (1982). “Modelling of photosynthetic response to environmental conditions” in Physiological plant ecology II. Water relations and carbon assimilation, eds. Lange O.L. Nobel P.S. Osmond C.B. Ziegler H., (Springer, Berlin, Germany) p. 551–87.

[B31] Fereres E. Soriano M.A. (2007). Deficit irrigation for reducing agricultural water use. *J. Expt*. Bot. 58, 147–159. 10.1093/jxb/erl165 17088360

[B32] Flexas J. Galmés J. Gall é, A. Gulías J. Pou A. Ribas-Carb ó, M.. (2010). Improving water use efficiency in grapevines: potential physiological targets for biotechnological improvement. Aust. J. Grape. Wine. R. 16, 106–121. 10.1111/j.1755-0238.2009.00057.x

[B33] Folimonova S.Y. Achor D.S. (2010). Early events of citrus greening (huanglongbing) disease development at the ultrastructural level. Phytopathol. 100, 949–958. 10.1094/PHYTO-100-9-0949 20701493

[B34] Foyer C.H. Noctor G. (2009). Redox regulation in photosynthetic organisms: signaling, acclimation, and practical implications. Antioxid. Redox. Sign. 11, 861–905. 10.1089/ars.2008.2177 19239350

[B35] Galvez D.A. Landhäusser S.M. Tyree M.T. (2011). Root carbon reserve dynamics in aspen seedlings: does simulated drought induce reserve limitation? Tree Physiol. 31, 250–257. 10.1093/treephys/tpr012 21444372

[B36] Genty B. Briantais J.M. Baker N.R. (1989). The relationship between the quantum yield of photosynthetic electron transport and quenching of chlorophyll fluorescence. Biochim.Biophys. Acta. 990, 87–92. 10.1016/S0304-4165(89)80016-9

[B37] Giannopolitis C.N. Ries S.K (1975). Superoxide dismutases. Annu. Rev.Biochem. 44, 147–159. 10.1146/annurev.bi.44.070175.001051 1094908

[B38] Gibon Y. Pyl E.T. Sulpice R. Lunn J.E. Höhne M. Günther M.. (2009). Adjustment of growth, starch turnover, protein content and central metabolism to a decrease of the carbon supply when *Arabidopsis* is grown in very short photoperiods. Plant Cell Environ. 32, 859–874. 10.1111/j.1365-3040.2009.01965.x 19236606

[B39] Graham J.H. Johnson E.G. Gottwald T.R. andIrey M.S. (2013). Presymptomatic fibrous root decline in citrus trees caused by huanglongbing and potential interaction with *Phytophthora* spp. Plant Dis. 97, 1195–1199. 10.1094/PDIS-01-13-0024-RE 30722426

[B40] Griffin K.L. Heskel M. (2013). Breaking the cycle: how light, CO_2_ and O_2_ affect plant respiration. Plant Cell Environ. 36, 498–500. 10.1111/pce.12039 23145557

[B41] Gudesblat G.E. Torres P.S. andVojnov A.A. (2009). Stomata and pathogensWarfare at the gates. Plant Signal. Behav. 4, 1114–1116. 10.4161/psb.4.12.10062 20514224 PMC2819434

[B42] Habermann G. Machado E.C. Rodrigues J.D. Medina C.L. (2003). CO_2_ assimilation, photosynthetic light response curves, andwater relations of ‘Pêra' sweet orange plants infected with *Xylella fastidiosa*. Braz. J. Plant Physiol. 15, 79–87. 10.1590/S1677-04202003000200003

[B43] Hamido S.A. Morgan K.T. Ebel R.C. Kadyampakeni D.M. (2017). Improved irrigation management of sweet orange with huanglongbing. Hort Sci. 52, 916–921. 10.21273/HORTSCI12013-17

[B44] Hamido S.A. Morgan K.T. Mahmoud K.A. (2016). Citrus huanglongbing impact on citrus trees biomass and nutrients uptake [Conference Presentation]. Annual soil science society conference. Division: Nutrient management and soil and plant analysis, Phoenix, AZ, United States. Available online at: https://scisoc.confex.com/scisoc/2016am/webprogram/Paper99487.html

[B45] Hanson J. Smeekens S. (2009). Sugar perception and signaling—an update. Curr. Opin. Plant Biol. 12, 562–567. 10.1016/j.pbi.2009.07.014 19716759

[B46] Harbinson J. Genty B. anf Baker N.R. (1990). The relationship between CO_2_ assimilation and electron transport in leaves. *Photosynth*. R. 25, 213–224. 10.1007/BF00033162 24420351

[B47] Heath R.L. Packer L. (1968). Photoperoxidation in isolated chloroplasts: I. Kinetics and stoichiometry of fatty acid peroxidation. Arch.Biochem.Biophys. 125, 189–198. 10.1016/0003-9861(68)90654-1 5655425

[B48] Hochberg U. Degu A. Fait A. andRachmilevitch S. (2013). Near isohydric grapevine cultivar displays higher photosynthetic efficiency and photorespiration rates under drought stress as compared with near anisohydric grapevine cultivar. *Physiol*. Plantarum 147, 443–452. 10.1111/j.1399-3054.2012.01671.x 22901023

[B49] Hong L. Chen C. Doddapaneni H. Duan Y. Civerolo E.L. Bai X.. (2010). A new diagnostic system for ultra-sensitive and specific detection and quantification of *Candidatus*Liberibacter asiaticus, the bacterium associated with citrus Huanglongbing. J. Microbiol. Meth. 81, 17–25. 10.1016/j.mimet.2010.01.014 20096734

[B50] Johnson E.G. Wu J. Bright D.B. Graham J.H. (2014). Root loss on presymptomaticHuanglongbing affected trees is preceded by *Candidatus*Liberibacter asiaticus root infection but not phloem plugging. Plant Pathol. 63, 290–298. 10.1111/ppa.12109

[B51] Kadyampakeni D.M. Morgan K.T. (2017). Irrigation scheduling and soil moisture dynamics influence water uptake by Huanglongbing affected trees. Sci. Hortic. 224, 272–279. 10.1016/j.scienta.2017.06.037

[B52] Kadyampakeni D.M. Morgan K.T. Schumann A.W. Nkedi-Kizza P. (2014). Effect of irrigation pattern and timing on root density of young citrus trees infected with Huanglongbing disease. HortTechnology 24, 209–221. 10.21273/HORTTECH.24.2.209

[B53] Kirschbaum M.U. Farquhar G.D. (1987). Investigation of the CO_2_ dependence of quantum yield and respiration in *Eucalyptus pauciflora*. Plant. Physiol. 83, 1032–1036. 10.1104/pp.83.4.1032 16665319 PMC1056496

[B54] Koh E.J. Zhou L. Williams D.S. Park J. Ding N. Duan Y.P.. (2012). Callose deposition in the phloem plasmodesmata and inhibition of phloem transport in citrus leaves infected with “*Candidatus*Liberibacter asiaticus”. Protoplasma 249, 687–697. 10.1007/s00709-011-0312-3 21874517

[B55] Kok B. (1948). A critical consideration of the quantum yield of Chlorella-photosynthesis. Enzymologial. 13, 1–56.

[B56] Kumar N. Kiran F. Etxeberria E. (2018). Huanglongbing-induced Anatomical Changes in Citrus Fibrous Root Orders. Hort Science. 53, 829–837. 10.21273/HORTSCI12390-17

[B57] Lei Y.B. Yin C.Y. Li C.Y. (2006). Differences in some morphological, physiological and biochemical responses to drought stress in two contrasting populations of *Populus przewalskii. Physiol*. Plant. 127, 182–191. 10.1111/j.1399-3054.2006.00638.x

[B58] Martinelli F. Dandekar A.M. (2017). Genetic mechanisms of the devious intruder candidatusliberibacter in *Citrus*. Front. Plant Sci. 8, 904. 10.3389/fpls.2017.00904 28620403 PMC5449717

[B59] Mattos-Jr D. Kadyampakeni D.M. da Silva J.R. Vashisth T. Boaretto R.M. (2020). Reciprocal effects of huanglongbing infection and nutritional status of citrus trees: a review. Trop.Plant Pathol. 45, 586–596. 10.1007/s40858-020-00389-y

[B60] Meltcafe D.B. Lobo-do-Vale R. Chaves M.M. Maroco J.P. Aragão L.E.O.C. Malhi Y.. (2010). Impacts of experimentally imposed drought on leaf respiration and morphology in an Amazon rain forest. *Funct*. Ecol. 24, 524–533. 10.1111/j.1365-2435.2009.01683.x

[B61] Murray M.G. Thompson W.F. (1980). Rapid isolation of high molecular weight plant DNA. Nucleic Acids Res. 8, 4321–4325. 10.1093/nar/8.19.4321 7433111 PMC324241

[B62] Nwugo C.C. Lin H. Duan Y. Civerolo E.L. (2013). The effect of ‘*Candidatus*Liberibacter asiaticus' infection on the proteomic profiles and nutritional status of pre-symptomatic and symptomatic grapefruit (*Citrus paradisi*) plants. BMC Plant Biol. 13, 59. 10.1186/1471-2229-13-59 23578104 PMC3668195

[B63] Panigrahi P. Sharma R.K. Hasan M. Parihar S.S. (2014). Deficit irrigation scheduling and yield prediction of ‘Kinnow' mandarin (*Citrus reticulate* Blanco) in a semiarid region. *Agric*. Water Manage. 140, 48–60. 10.1016/j.agwat.2014.03.018

[B64] Pedroso F.K.J.V. Prudente D.A. Bueno A.C.R. Machado E.C. Ribeiro R.V. (2014). Drought tolerance in citrus trees is enhanced by rootstock-dependent changes in root growth and carbohydrate availability. Environ. Exp. Bot. 101, 26–35. 10.1016/j.envexpbot.2013.12.024

[B65] Pérez-Pérez J.G. Robles J.M. Botía P. (2009). Influence of deficit irrigation in phase III of fruit growth on fruit quality in “lane late” sweet orange. *Agric*. Water Manag. 96, 969–974. 10.1016/j.agwat.2009.01.008

[B66] Pinheiro C. Chaves M.M. (2011). Photosynthesis and drought: can we make metabolic connections from available data? J. Exp. Bot. 62, 869–882. 10.1093/jxb/erq340 21172816

[B67] Pons T.L. Welschen R.A.M. (2002). Overestimation of respiration rates in commercially available clamp-on leaf chambers. Complications with measurement of net photosynthesis. Plant Cell Environ. 25, 1367–1372. 10.1046/j.1365-3040.2002.00911.x

[B68] Puglisi I. Nicolosi E. Vanella D. Lo Piero A.R. Stagno F. Saitta D.. (2019). Physiological and biochemical responses of orange trees to different deficit irrigation regimes. Plants 8, 423. 10.3390/plants8100423 31627476 PMC6843479

[B69] Ramel F. Sulmon C. Gouesbet G. Couee I. (2009). Natural variation reveals relationships between pre-stress carbohydrate nutritional status and subsequent responses to xenobiotic and oxidative stress in Arabidopsis thaliana. *Ann*. Bot. 104, 1323–1337. 10.1093/aob/mcp243 19789177 PMC2778391

[B70] Revanna R. Turnbull M.H. Shaw M.L. Wright K.M. Butler R.C. Jameson P.E.. (2013). Measurement of the distribution of non-structural carbohydrate composition in onion populations by a high-throughput microplate enzymatic assay. J. Sci. Food Agric. 93, 2470–2477. 10.1002/jsfa.6062 23494930

[B71] Ribeiro R.V. Machado E.C. Santos M.G. Oliveira R.F. (2009). Photosynthesis and water relations of well-watered orange plants as affected by winter and summer conditions. Photosynthetica 47, 215–222. 10.1007/s11099-009-0035-2

[B72] Romero-Conde A. Kusakabe A. Melgar J.C. (2014). Physiological responses of citrus to partial rootzone drying irrigation. Sci.Hortic. 169, 234–238. 10.1016/j.scienta.2014.02.022

[B73] Salazar-Parra C. Aranjuelo I. Pascual I. Erice G. Sanz-Sáez A. Aguirreolea J.. (2015). Carbon balance, partitioning and photosynthetic acclimation in fruit-bearing grapevine (*Vitis vinifera* L. cv. Tempranillo) grown under simulated cli- mate change (elevated CO_2_, elevated temperature and moderate drought) scenarios in temperature gradient greenhouses. J Plant Physiol. 174, 97–109. 10.1016/j.jplph.2014.10.009 25462972

[B74] Scholander P.F. Hammel H.T. Hemingsen E.A. Bradstreet E.D. (1964). Hydrostatic pressure and osmotic potential of leaves of mangrove and some other plants. *Proc. Natl. Acad. Sci*. U.S.A. 52, 119–125. 10.1073/pnas.52.1.119 16591185 PMC300583

[B75] Shapiro J.B. Griffin K.L. Lewis J.D. Tissue D.T. (2004). Response of *Xanthium strumarium* leaf respiration in the light to elevated CO_2_ concentration, nitrogen availability and temperature. New Phytol. 162, 377–386. 10.1111/j.1469-8137.2004.01046.x

[B76] Shu S. Guo S.R. Sun J. Yuan L.Y. (2012). Effects of salt stress on the structure and function of the photosynthetic apparatus in *Cucumis sativus* and its protection by exogenous putrescine. Physiol. Plantarum 146, 285–296. 10.1111/j.1399-3054.2012.01623.x 22452600

[B77] Silva J.R. Alvarenga F.V. Boaretto R.M. Lopes J.R.S. Quaggio J.A. Coletta Filho H.D.. (2020). Following the effects of micronutrient supply in HLB-infected trees: plant responses and ‘*Candidatus*Liberibacter asiaticus' acquisition by the Asian citrus psyllid. Trop. Plant Pathol. 45, 597–610. 10.1007/s40858-020-00370-9

[B78] Smirnova J. Fernie A.R. Spahn C.M.T. Steup M. (2017). Photometric assay of maltose and maltose-forming enzyme activity by using 4-alpha-glucanotransferase (DPE2) from higher plants. Anal.Biochem. 532, 72–82. 10.1016/j.ab.2017.05.026 28576440

[B79] Smith A.M. Zeeman S.C. (2006). Quantification of starch in plant tissues. Nat. Protoc. 1342, 3. 10.1038/nprot.2006.232 17406420

[B80] Stetler M. Eicke S. Mettler T. Messerli G. Hortensteiner S. Zeeman S.C. (2009). Blocking the metabolism of starch breakdown products in *Arabidopsis* leaves triggers chloroplast degradation. *Mol*. Plant. 2, 1233–1246. 10.1093/mp/ssp093 19946617 PMC2782796

[B81] Strasser B.J. Strasser RJ (1995). Measuring fast fluorescence transients to address environmental questions: The JIP-test. In: Photosynthesis: from light to biosphere, ed Mathis P. (Kluwer, Dordrech) p. 977–980.

[B82] Strasser R.J. Srivastava A. Govindjee (1995). Polyphasic chlorophyll *a* fluorescence transient in plants and cyanobacteria. *Photochem*. Photobiol. 61, 32–42. 10.1111/j.1751-1097.1995.tb09240.x

[B83] Strasser R.J. Tsimilli-Michael M. Srivastava A. (2004). “Analysis of the chlorophyll fluorescence transient” in: Chlorophyll fluorescence: a signature of photosynthesis, advances in photosynthesis and respiration, eds Papageorgiou G.C. Govindjee (Springer, Dordrecht) 321–362. 10.1007/978-1-4020-3218-9_12

[B84] Strauss A.J. Krüger G.H.J. Strasser R.J. Van Heerden P.D.R. (2006). Ranking of dark chilling tolerance in soybean genotypes probed by the chlorophyll *a* fluorescence transient O-J- I-P. *Environ. Exp*. Bot. 56, 147–157. 10.1016/j.envexpbot.2005.01.011

[B85] Thalmann M. Pazmino D. Seung D. Horrer D. Nigro A. Meier T.. (2016). Regulation of Leaf Starch Degradation by Abscisic Acid Is Important for Osmotic Stress Tolerance in Plants. The Plant Cell. 28, 1860–1878. 10.1105/tpc.16.00143 27436713 PMC5006701

[B86] Villar R. Held A. A. Merino J. (1994). Comparison of methods to estimate dark respiration in the light in leaves of two woody species. Plant Physiol. 105, 167–172. 10.1104/pp.105.1.167 12232196 PMC159342

[B87] Viola R. Davies H.V. (1992). A microplate reader assay for rapid enzymatic quantification of sugars in potato tubers. Potato Res. 35, 55–58. 10.1007/BF02357723

[B88] von Caemmerer S. Farquhar G.D. (1981). Some relationships between the biochemistry of photosynthesis and the gas exchange of leaves. Planta 153, 376–387. 10.1007/BF00384257 24276943

[B89] Wang N. Trivedi P. (2013). Citrus huanglongbing: a newly relevant disease presents unprecedented challenges. Phytopathol. 103, 652–665. 10.1094/PHYTO-12-12-0331-RVW 23441969

[B90] Wingler A. Quick W.P. Bungard R.A. Bailey K.J. Lea P.J. andLeegood R.C. (1999). The role of photorespiration during drought stress: an analysis utilizing barley mutants with reduced activities of photorespiratory enzymes. Plant Cell Environ. 22, 361–373. 10.1046/j.1365-3040.1999.00410.x

[B91] Zeeman S.C. Smith S.M. Smith A.M. (2004). The breakdown of starch in leaves. New Phytol. 163, 247–261. 10.1111/j.1469-8137.2004.01101.x 33873622

[B92] Zlatev Z. (2009). Drought-induced changes in chlorophyll fluorescence of young wheat plants. Biotechnol. 23, 437–441. 10.1080/13102818.2009.10818458

