# Supplementary material for: Effects of Deficit Irrigation and Huanglongbing on Sweet Orange Trees
Source: Front Plant Sci. 2021 Oct 15;12:731314. doi: 10.3389/fpls.2021.731314 (PMC8554030; doi:10.3389/fpls.2021.731314)
Supplement: Supplementary file 1 [file Data_Sheet_1.docx]

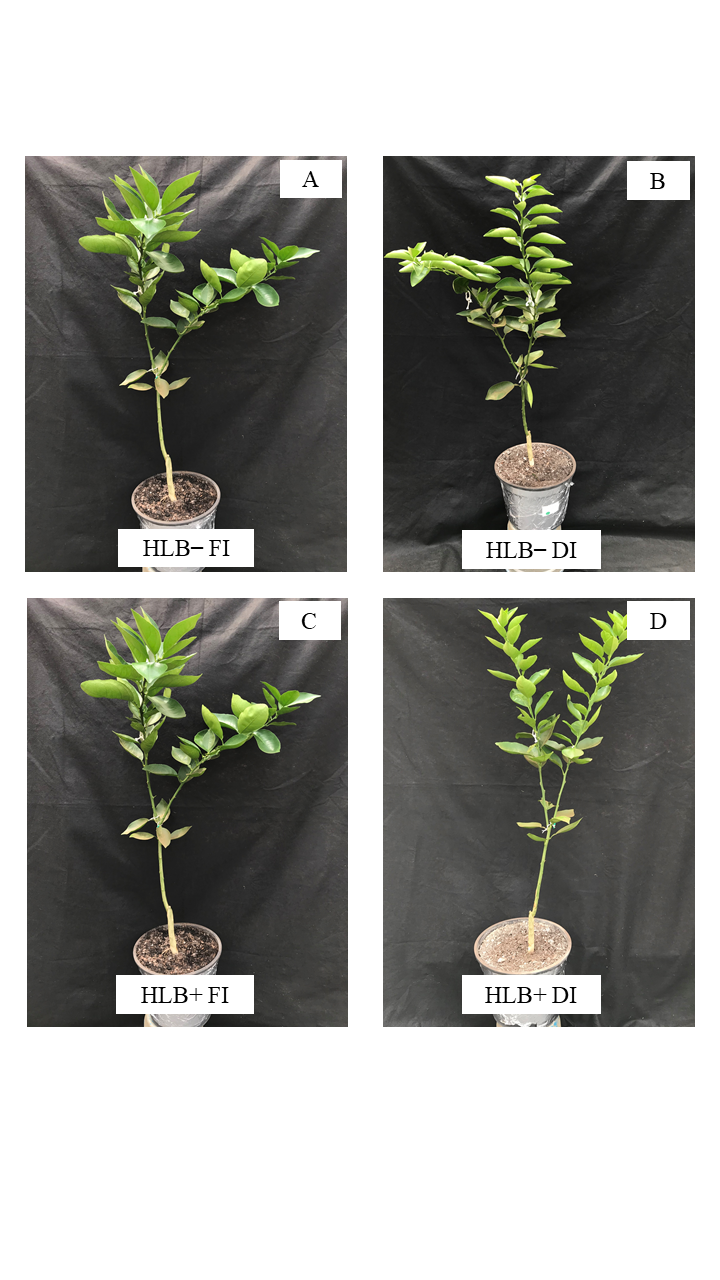


**Supplementary Figure S1.** Phenotipic images of ‘Valência’ orange trees [*Citrus sinensis* (L.) Osbeck] grafted onto citrumelo ‘Swingle’ rootstock [*Citrus paradisi* Macfad. x *Poncirus trifoliata* (L.) Raf.] either infected with ‘*Ca*. Liberibacter asiaticus’ (+) or healthy (–) and exposed to two water management treatments – full-irrigated (FI) and deficit irrigated (DI).
