# Supplementary material for: Effects of Deficit Irrigation and Huanglongbing on Sweet Orange Trees
Source: Front Plant Sci. 2021 Oct 15;12:731314. doi: 10.3389/fpls.2021.731314 (PMC8554030; doi:10.3389/fpls.2021.731314)

**Supplementary Figure S2.** Phenotipic images of roots of citrumelo ‘Swingle’ rootstock [*Citrus paradisi* Macfad. x *Poncirus trifoliata* (L.) Raf.] either infected with ‘*Ca*. Liberibacter asiaticus’ (+) or healthy (–) and exposed to two water management treatments – full-irrigated (FI) and deficit irrigated (DI).


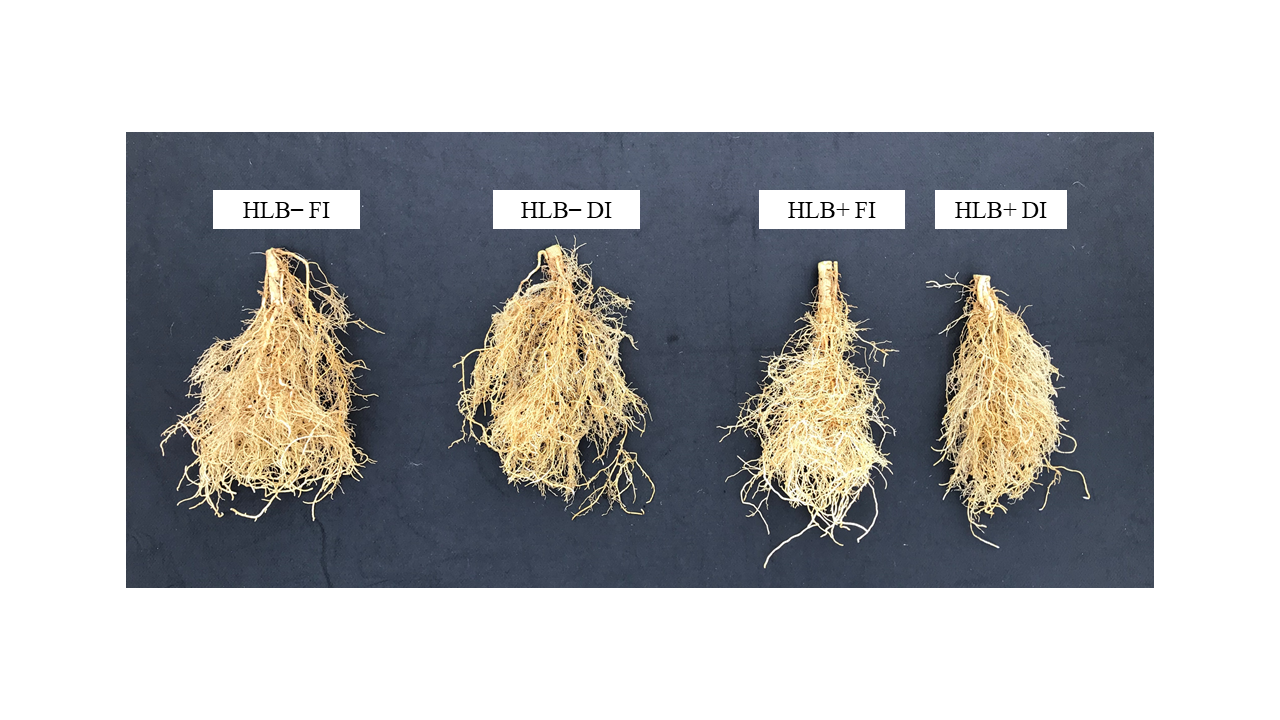

Supplement: Supplementary file 2 [file Data_Sheet_2.docx]
